# Insights on a Hierarchical MFI Zeolite: A Combined Spectroscopic and Catalytic Approach for Exploring the Multilevel Porous System Down to the Active Sites

**DOI:** 10.1021/acsami.1c11614

**Published:** 2021-09-20

**Authors:** Alessia Airi, Matteo Signorile, Francesca Bonino, Pierluigi Quagliotto, Silvia Bordiga, Johan A. Martens, Valentina Crocellà

**Affiliations:** † Department of Chemistry, NIS and INSTM Reference Centre, University of Turin, Via G.Quarello 15/A 10135 and Via P.Giuria 7, 10125 Turin, Italy; ‡ Centre for Surface Chemistry and Catalysis, KU Leuven, Celestijnenlaan 200F, Box 2461, B-3001 Leuven, Belgium

**Keywords:** hierarchical MFI, multilevel porosity, textural properties, IR and Raman spectroscopies, acidic active sites, *n*-decane hydroconversion

## Abstract

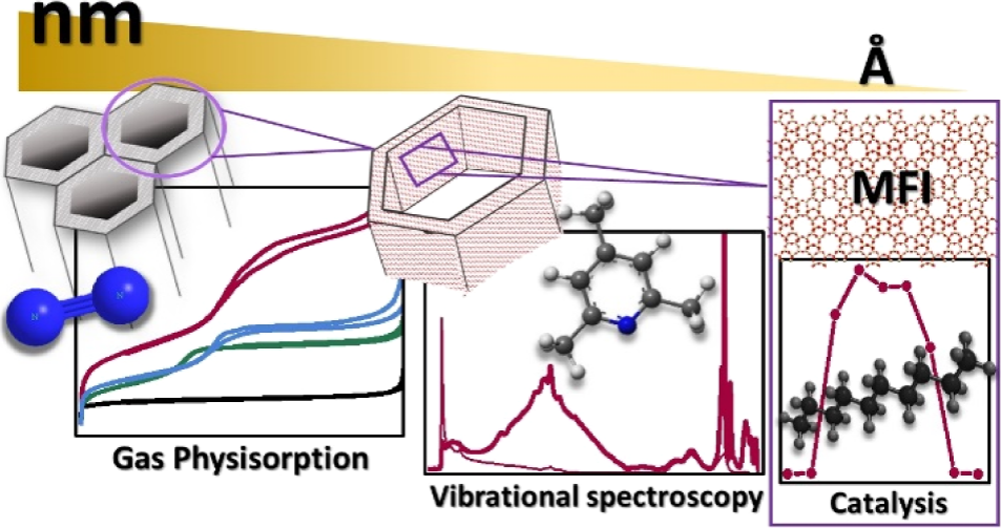

The hierarchization of zeolites to overcome the major drawbacks related to molecular diffusion limitation in micropores is a popular concept in heterogeneous catalysis. Despite the constant increase of new synthesis strategies to produce such hierarchical systems, the deep knowledge of their structural arrangement and how the zeolitic lattice is organized in a multilevel porous system is often missing. This information is essential to design a structure, tuning the porosity and the distribution of easily accessible active sites, and successively controlling the catalytic properties. In the present work, the synthesis of one of the most sophisticated forms of the hierarchical ZSM-5 zeolite has been reproduced, obtaining two multilevel porous materials with different crystallinity degrees, with the final aim of investigating and clarifying the finest features of their active sites. For this purpose, an extended characterization step by means of a unique multitechnique approach has been performed, thus revealing the active site nature, abundance, and distribution. IR spectroscopy with different molecular probes and a targeted catalytic test based on the hydroconversion reaction of *n*-decane were the toolbox for disclosing how the MFI lattice takes part in the hierarchical structure and how it, working in synergy with the mesoporous system, confers to this material a totally new shape–size selectivity. Merging the information obtained for the synthesized hierarchical zeolite with the characterization results of two reference materials (a mesoporous aluminum-containing MCM-41 and a microporous commercial ZSM-5), it was possible to define an internal and external map of the pore network of this complex and unique molecular sieve, where strong Brønsted acidic sites are located at the mouth of the MFI micropores and, at the same time, exposed at the surface of the mesoporous channels. Hence, the possibility of easily releasing bulky products is ensured and the application possibilities of the MFI lattice are expanded beyond cracking reactions.

## 1 Introduction

The rigidity and the precise shape of the inner space of zeolites, organized in a microporous channel system, combined with the distribution of the active sites, allow them to be classified as molecular sieves and are responsible for their shape-selectivity in catalysis.^1,2^ These characteristics, indeed, allow the control and prediction of the access of reactants to the active sites and drive the formation of selected products through the imposition of the shape of intermediates and transition states. At the same time, the zeolites’ active sites confinement in a narrowed space represents the major drawback of these materials. Indeed, the zeolite microchannels (diameter < 1 nm) cause strong limitations in the accessibility and in the diffusion rate of the reacting species and force the molecular transport, giving rise to the catalyst deactivation by obstruction. For this reason, mesoporous silicate materials obtained from synthetic design inspired by zeolites, such as MCM-41^3^ and SBA-15,^4^ attract considerable interest because they offer increased surface areas and a reduced deactivation rate. On the other hand, the amorphous nature of such materials does not allow them to compete with zeolitic microcrystalline systems in terms of stability during the reactions, shape selectivity, and controllability of the active sites.^5^ Therefore, the most promising and innovative route is the production of systems that would preserve the crystalline arrangement and the bulk properties of a zeolite, while being a part of a multilevel structured system of pores with different diameters, the so-called hierarchical zeolites.^6,7^ Indeed, the existence of a mesoporous network allows increasing the accessible surface area and the mass transport, limiting possible deactivation phenomena, whereas the ordered microporous domains offer a shorter path for reagent diffusion, maintaining the well-known shape selectivity of the zeolitic systems. Such a conformation paves the way for a wide range of possible substrates and specific products determined using a multilevel molecular sieve.^8^

The routes for introducing additional porous levels into an ordered material are substantially distinguishable in two main approaches: “top-down” and “bottom-up”. The “top-down” strategies refer essentially to postsynthetic treatments over crystalline zeolites, in order to promote the formation of mesopores, such as partial desilication or dealumination.^9,10^ Contrarily, “bottom-up” approaches imply the growing of a hierarchical system directly during synthesis, through the application of proper structure-directing agents (SDA), which drive the atomic assembly of the framework.^11−14^ As a result of this new fruitful line of synthetic research, a large amount of new materials presenting both micro and meso (or macro) pores are documented, but, when they can actually be considered “hierarchical zeolites” remains a matter of discussion. The latter point is related to the term “hierarchical” itself, often used in a broad sense. Hierarchy in a porous system, in fact, implies a continuum between the different pore levels, passing through a subdivision of the inner space by distinct, but complementary, structural units: the channels must be connected without an interruption, like the organization of the vascular system of the human body.^15^ This complex structure affects and defines the catalyst performances in a shape-size-driven reaction pathway. Materials presenting segregated regions with different porosities or non-connected pores of various dimensions cannot actually be considered hierarchical, even if they present multiple levels of pore diameters; this type of structures can be usually obtained through desilication by alkaline hydroxides, a not well-controllable process.^16,17^ The discrimination of an actual hierarchical system by means of common characterization techniques is not always straightforward. For example, the structure resolution by powder X-ray diffraction (PXRD) is often not possible due to the reduced extension of the crystalline domains in micro–mesoporous systems.^18,19^ Therefore, the identification of a “truly” hierarchical zeolite requires a tailor-made protocol for characterization.

Successful attempts with the creation of mesoporous zeolites have been achieved in 2006 by applying the quaternary ammonium surfactant in combination with cationic polymers,^22^ and then afterward by developing hard templating techniques.^21^ In 2011,^20^ Ryoo and co-workers supplied a revolutionary synthetic approach for the soft templated formation of mesoporous zeolites, based on the bifunctional action of a single-molecular SDA. The bifunctional templating agent drives the assembly of the silicate framework over the surface of the micelles formed in aqueous medium, producing, at the same time, both micro- (by means of quaternary ammonium functionalities) and mesopores (by means of long alkylic tails). They proposed a synthesis templated by the dual surfactant C_18_–N_3_–C_18_ (C_18_ refers to the length of the alkylic chains and N_3_ to the number of ammonium functionalities) of an aluminum silicate organized in hexagonal mesoporous channels (similar to the MCM-41), encompassed by zeolitic structures (i.e., thin crystalline zeolitic walls with a MFI topology). In the same paper,^20^ Ryoo and co-workers reported this new molecular sieve to be highly active as a catalyst for various acid-catalyzed reactions of bulky molecular substrates, compared with conventional zeolites and ordered mesoporous amorphous materials. In this scenario, it is evident how the considerably improved catalytic activity of this hierarchically structured zeolite deserves a further specialized investigation to clarify the finest features of its active sites.

For this reason, in the present work, we decided to replicate the synthesis procedure proposed by Ryoo to develop a tailor-made advanced characterization procedure for disclosing the nature, abundance, and distribution of the catalyst active sites, highlighting how the peculiar hierarchical organization affects the shape-size selectivity of the MFI framework. After the synthesis of the catalyst in its acidic form, we retraced the structural characterization of the material by means of PXRD and transmission electron microscopy (TEM), and then we investigated the active sites’ intimate nature and location within the multilevel porous system, by combining a unique spectroscopic approach and targeted catalytic experiments. For this purpose, after a careful analysis of the size and volume of the different pore families by applying the non-localized density functional theory (NLDFT) model to nitrogen physisorption isotherms, different molecules with various steric hindrance and basic characters have been selected as probes.^23−26^ Such information has been merged with the results obtained by employing the material as the catalyst in *n*-decane hydroconversion. The so-called “decane test” represents a powerful “characterization” technique for disclosing the inner space of a zeolitic material,^27^ through the evaluation of isomerization and cracking products during the conversion pathways. For comparison, the same multitechnique characterization approach has been carried out on two reference materials (an aluminum containing MCM-41 as a mesoporous model and a commercial ZSM-5 as a microporous reference) finally allowing the indirect evaluation of the internal and external map of the porous network of this complex and unique molecular sieve.

## 2 Experimental Section

### 2.1 Commercial Reference ZSM-5 CBV3024E (Si/Al = 15)

The zeolite in the ammonic form was provided by Zeolyst and converted into the protonic form by thermal treatment at high temperature (500 °C in vacuum) when needed. The protonic acid zeolite is hereafter referred to as H-ZSM-5.

### 2.2 Synthesis

#### 2.2.1 Synthesis of Reference Al-MCM-41 (Nominal Si/Al = 20)

The mesoporous silicate Al-MCM-41 was synthetized by hydrothermal treatment, according to the procedure published by Vaschetto et al. in 2013.^28^ Tetraethyl orthosilicate (TEOS, Sigma-Aldrich, ≥99.0%) was used as a silicon source, sodium aluminate (NaAlO_2_ Sigma-Aldrich, technical, anhydrous) as an aluminum precursor, and hexadecyltrimethylammonium bromide (CTA-Br, Sigma-Aldrich, 95%) as an organic template. The gel composition was chosen following the molar ratios
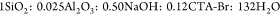


First, 0.589 g NaOH (Sigma-Aldrich, ≥98%, pellets, anhydrous) is solubilized in 70 mL H_2_O within a two-neck round-bottom flask, under stirring at 40 °C, then 1.288 g CTA-Br is added, followed by dropwise addition of 6.58 mL TEOS. The aqueous solution is kept under stirring for 30 min at constant temperature. After this time, 0.1207 g NaAlO_2_ is added and the mixture is stirred for 4 h at room temperature, to promote the formation of the gel. At the end, the solution is kept in a teflon-coated stainless-steel autoclave for the hydrothermal treatment at a constant temperature of 100 °C for 2 days. The material is then separated by filtration, washed with deionized water, and then calcined in a tubular oven within an alumina crucible, heating up to 550 °C (with a ramp of 2 °C/min) under N_2_ flow and then O_2_ flow at the same temperature for 7 h.

#### 2.2.2 Synthesis of Non-Commercial Surfactant C_18_–N_3_–C_18_

The molecule has been synthetized following the procedure of Na et al.^20^ The first intermediate is obtained by mixing 5 g *N*,*N*-dimethyloctadecan-1-amine (TCI Chemicals, >90.0%) and 25.8 mL 1,6-dibromohexane (TCI Chemicals, >97.0%) (1:10 ratio) then solubilized dropwise (through a drip funnel) within a 1:1 mixture of 250 mL acetonitrile (Sigma-Aldrich, ≥99.9%) and 250 mL toluene (Sigma-Aldrich, ≥99.5%). The reaction is carried forward for 24 h under stirring at 65 °C. At the end of the reaction, the solvents are removed by evaporation under vacuum, obtaining an oily raw product. The product is then crystallized and washed from reactant residues by ethyl ether baths (400 mL), under stirring for 2 h and repeating the procedure three times. A white precipitate was obtained, isolated by in vacuum filtration. For the second intermediate, 10 g 1-bromooctadecane (TCI Chemicals, >97.0%) and 64.1 mL *N*1,*N*1,*N*6,*N*6-tetramethyl hexane-1,6-diamine (TCI Chemicals >98.0%) (1:10 ratio) were reacted following the same procedure described for the intermediate 1. The final product is obtained by the coupling of intermediates 1 and 2 in a ratio of 1:1 and solubilized dropwise in 500 mL acetonitrile at 65 °C. The reaction is conducted under stirring and monitored by frequent sampling and analysis by mass spectrometry. All the products have been analyzed by ^1^H NMR spectroscopy, confirming the success of the synthesis.

#### 2.2.3 Synthesis of Hierarchical mM-Z (Nominal Si/Al = 20)

The hierarchical aluminum silicate material, hereafter referred to as mM-Z (microMeso Zeolite), has been synthesized by hydrothermal treatment, using the non-commercial surfactant C_18_–N_3_–C_18_ as the templating agent, following the procedure of Na et al.^20^

The gel composition is as follows
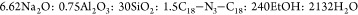


Two different samples have been obtained and described here, evaluating the influence of different temperature control during the gel-aging process.

The sample hereafter termed mM-Z1 has been obtained as follows: first C_18_–N_3_–C_18_ is solubilized in 4.639 mL ethanol (Sigma-Aldrich, ≥99.5%) and 6.14 mL deionized water and kept under stirring at 65 °C until obtaining a clear liquid, with the formation of surfactant micelles. Then, 0.0478 g NaAlO_2_ and 0.1480 g NaOH are solubilized in 6 mL of water, where subsequently, 2.218 g TEOS has been added. This second solution is added dropwise to the first one and then kept for 6 h under stirring at a liquor temperature of 65 °C for the gel aging. During this phase, the temperature has been monitored by immersing a traditional mercury thermometer directly into the reaction liquid. This ensures that the temperature remains constant throughout this stage prior to hydrothermal treatment.

The sample hereafter labeled mM-Z2 has been obtained with the same procedure, but with a different temperature control. During surfactant micellization and gel aging, the temperature has been kept at 60 °C by electronic control of the heating bath through the thermocouple integrated in the heating system. It is assumed that there is a deviation of about 10 °C less from the temperature of the reaction liquid. In both cases, the white gels were then kept in a teflon-lined steal autoclave for the hydrothermal treatment, in an oven at 140 °C for 8 days under tumbling conditions. At the end, the obtained powders were filtered under vacuum and repeatedly washed with deionized water, then calcined within an alumina crucible, and kept in a tubular oven at 550 °C (a heating ramp of 2 °C/min) for 7 h under dry air flow.

#### 2.2.4 Cation Exchange

mM-Z1, mM-Z2, and Al-MCM-41 have been synthesized in the sodic form. The Na^+^ cations were exchanged with NH_4_^+^ ions to generate the acidic catalyst. The procedure adopted is as follows: the powder is immersed for 6 h in a 1 M solution of NH_4_NO_3_ (Sigma-Aldrich, ≥99.0%) (20 mL solution/g sample) at 80 °C then the ion-exchanged material is separated by centrifugation and the same procedure is repeated three times. NH_4_^+^ substitutes Na^+^ in the aluminum silicate structure, and finally NH_3_ is released, when needed, by thermal activation (refer to this in the further sections), at high temperature (400–500 °C), leaving only H^+^ for charge balancing, giving the final material with acidic character. The samples in the protonic form are labeled as: H-mM-Z1, H-mM-Z2, and H-Al-MCM-41.

### 2.3 Characterization Techniques

#### 2.3.1 Transmission Electron Microscopy

The TEM analysis has been conducted using a TEM JEOL JEM 3010 UHR microscope (a theoretical resolution of 0.17 nm) equipped with a LaB_6_ electron source working at 300 kV of accelerating potential and an EDS OXFORD X-STREAM energy dispersion detector. The images have been collected using a CCD camera Gatan, model 894 US1000 (2k × 2k). The sample has been prepared depositing a small amount of powder over a 200-mesh lacey carbon copper grid.

#### 2.3.2 X-ray Powder Diffraction (PXRD)

The XRD measurements have been carried out using the Bragg–Brentano geometry with a PANalytical PW3050/60 X’Pert PRO MPD diffractometer with a Cu anode (Kα = 1.5418 Å) and an X’Celerator detector.

#### 2.3.3 N_2_ Physisorption

Isothermal N_2_ physisorption measurements at liquid nitrogen temperature were performed on a Micromeritics ASAP 2020. Prior to the measurement, the powders were degassed overnight at 100 °C and 4 h at 350 °C. Specific surface areas were determined by using both the Brunauer–Emmett–Teller and the Langmuir models. Pore size distributions were obtained by applying the NL-DFT method.

#### 2.3.4 Infrared Spectroscopy

##### 2.3.4.1 Transmission IR Experiments

The samples were analyzed in the form of self-supporting pellets kept in a home-made quartz cell with KBr or CaF_2_ windows (depending on the molecular probe used), designed for thermal outgassing before each experiment. The thermal activation consisted of the controlled heating at 5 °C/min until 500 °C, with the contemporary removal of gaseous species through a glass-line equipped with vacuum pumps, until reaching 5 × 10^–4^ mbar of internal pressure of the quartz cell. Then, in order to oxidize all the adsorbed species on the porous sample surface, 100 mbar of pure O_2_ was introduced in the cell and kept in contact for 1 h. Finally, O_2_ was outgassed, and the sample was cooled to RT, under vacuum. The infrared (IR) spectra were acquired in transmission mode with a Bruker Vertex 70 spectrophotometer equipped with a MCT cryodetector, collecting 32 scans for each spectrum with a resolution of 2 cm^–1^. Under acquisition, the samples were kept connected to the vacuum glass-line, which allowed the dosage of gaseous probe molecules for in situ measurements, recording the spectral changes during adsorption/desorption. Every probe molecule—carbon monoxide (CO);^29^ pyridine (Py);^30^ and 2,4,6-trimethyl pyridine (collidine)—^31^ requires a specific experimental setting, according to the specific literature reported.^32,33^

##### 2.3.4.2 ATR-IR Experiments

The spectra were collected on the same instrument, opportunely equipped with a Bruker Platinum ATR accessory with a diamond single-reflection internal refraction element and a DTGS detector. The samples have been analyzed without any previous treatment.

#### 2.3.5 Raman Spectroscopy

The 244 nm Raman spectra were collected on a Renishaw inVia Raman microscope spectrometer, equipped with a Coherent Innova 300C motoFreD frequency-doubled Ar^+^ laser as the excitation source, a 3600 line/mm grating, and a UV-enhanced CCD detector. The light was focused on samples through a 15× objective. In order to prevent degradation phenomena induced by the intense, highly energetic excitation light, samples were kept under continuous rotation by using a home-made sample holder.^34^ Three spectra were collected for each sample and averaged to yield a better signal-to-noise ratio.

### 2.4 Catalytic Hydroconversion of *n*-Decane

The samples were first loaded with 0.3% (w/w) of Pt, by wet impregnation with an aqueous solution of Pt(NH_3_)_4_Cl_2_ (Sigma-Aldrich, 98%). Once dried, the powders were pelletized and kept in a high-throughput reactor, a home-made design of KU Leuven University.^35^ In each experiment, 50 mg of bifunctional catalyst pellets was loaded in quartz reactor tubes with an internal diameter of 2 mm. The materials have been pretreated in situ by heating to 400 °C at 5 °C/min under O_2_ flow for 1 h, then flushed with N_2_ for 25 min, and finally reduced in a pure H_2_ atmosphere at 400 °C for 1 h. Before starting the feed *n*-decane injection, the reactor has been cooled down to 100 °C. The H_2_/*n*-decane molar ratio in the catalytic experiments was 214, and the space time (*W*/*F*_o_) was 1400 kg s/mol. The reaction temperature was increased stepwise at constant space time. The reaction products were sampled for in-line gas chromatographic (GC) analysis 1 h after reaching a temperature set point with a rising slope of 5 °C for every step until complete conversion of the feed *n*-decane was reached.

## 3 Results and Discussion

Herein, we report the complete, tailor-made characterization protocol of the two hierarchical mM-Z materials, with the aim of disclosing their intimate and finest features. The two materials exhibit non-negligible differences, which will be described in detail in the following. This fact shows that slight variations in the synthesis procedure, and in particular during the aging phase prior to hydrothermal treatment, have a strong influence on the final material.

From ICP measurements, compared also with EDX elemental analysis, the average silicon to aluminum (SAR) ratio of the mM-Z samples is equal to 15. The two reference materials (the mesoporous MCM-41 and the commercial microporous ZSM-5) have the same SAR of the hierarchical structures.

### 3.1 Morphological and Structural Properties

**Figure 1:**
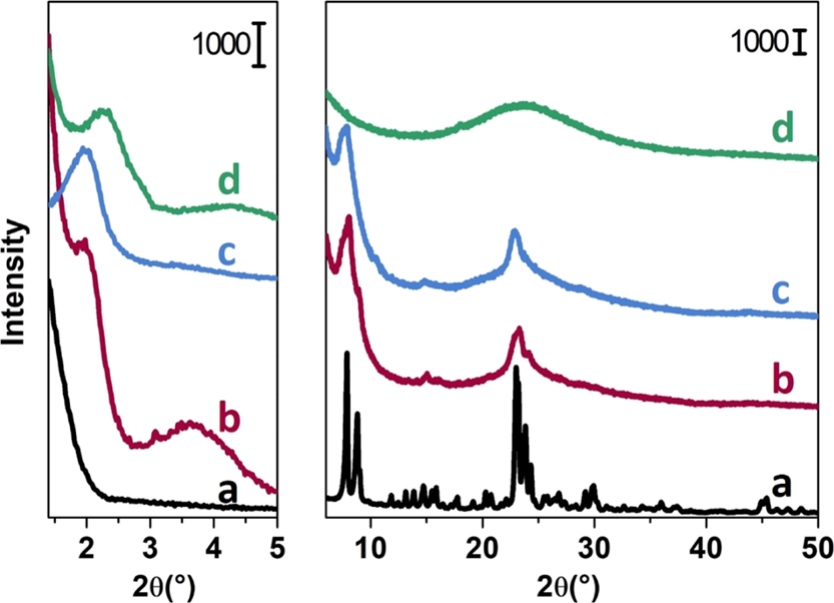
Left panel: small angles. Right panel: wide-angle powder XRD patterns of calcined samples: reference ZSM-5 (a), mM-Z1 (b), mM-Z2 (c), and Al-MCM-41 (d).

Figure 1 shows the PXRD patterns of mM-Z1 (b) and mM-Z2 (c), compared with the bulk commercial ZSM-5 (a) and the Al-MCM-41 sample (d), both in the low-angle (below 5°2θ) and wide-angle regions. The mM-Z1 sample displays two resolved Bragg reflections at around 2 and 3.5° (see the Figure 1 left panel). These peaks are totally absent in the MFI pattern and they have been indicized by Na et al.^20^ as (*hk*) reflections, corresponding to a long distance *d* of a mesoscale lattice with 2D hexagonal symmetry, similar to MCM-41.^36^ The reflections (11) and (10) are well visible for mM-Z1, whereas only the (10) peak is present in the mM-Z2 diffractogram. In Figure 1 right panel (wide angles), both mM-Z samples exhibit only the principal diffraction peaks corresponding to the reflections of the MFI lattice along the [a*] crystallographic direction. As explained by Ryoo et al.,^20^ determining the microporous framework structure of such materials accurately by XRD is challenging because the mesopore walls are composed of only a single layer of zeolitic micropores, which can be less than a single-unit-cell dimension of a bulk zeolite. Moreover, the zeolite-like mesopore walls extend over a very narrow diffractive domain in width. The broader peaks of both mM-Z samples indicate the less extension of the crystalline domains compared to the bulk reference H-ZSM-5 zeolite. The mM-Z1 sample presents more resolved peaks with respect to mM-Z2, suggesting the presence of more structural elements of the MFI lattice in its mesoporous framework. Observing the profile of both mM-Z diffractograms, which present a limited number of rather wide reflections, it is possible to exclude the presence of a segregated MFI phase, but at the same time, the baseline trend does not suggest the presence of a significant amorphous phase. The hierarchical samples, therefore, have their own level of order, characterized by thin crystalline domains. The second level of order is detectable by the XRD reflections at small angles, the trace of a larger structure, comparable to the 2D hexagonal openings of MCM-41 (d).

**Figure 2:**
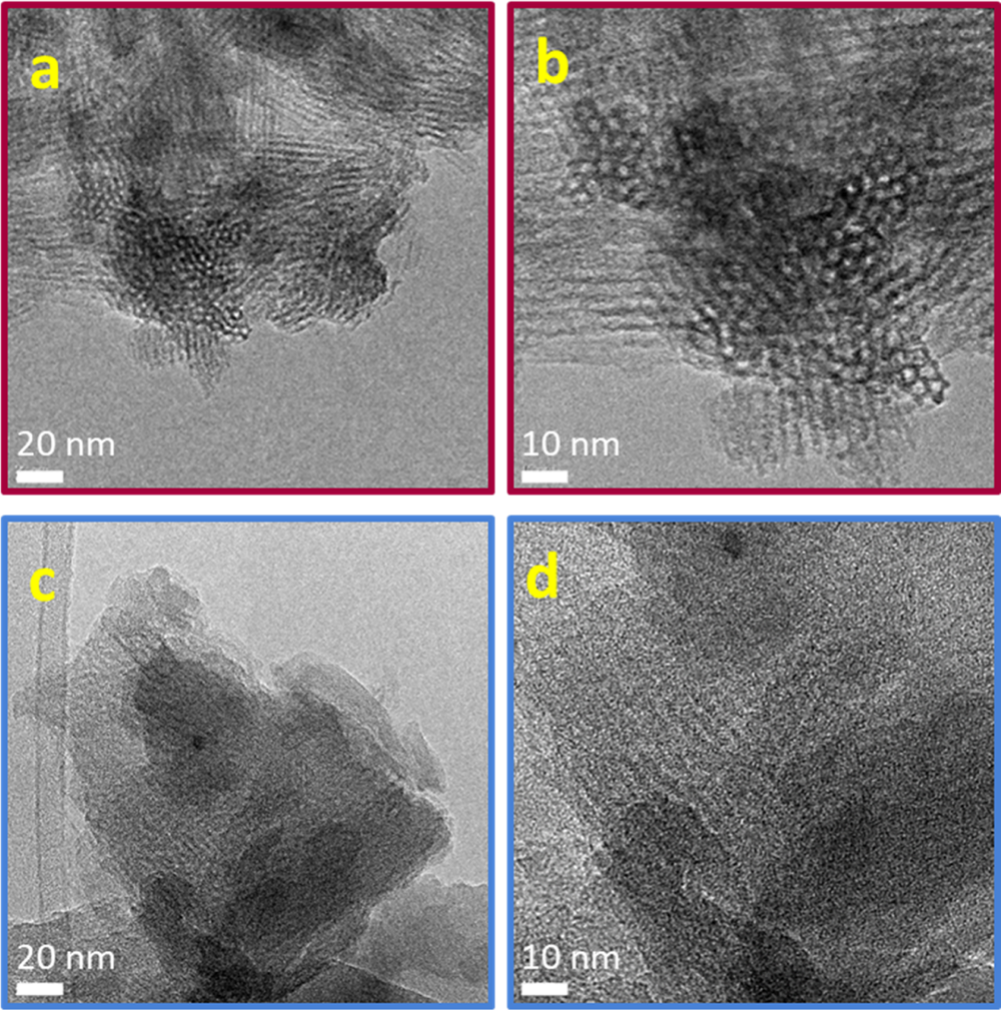
TEM micrographs of calcined mM-Z1 (a,b) and calcined mM-Z2 (c,d) at increasing magnifications.

The TEM micrographs shown in Figure 2 confirm the presence of a hexagonal array of mesopores of around 4 nm for mM-Z1 (Figure 2a,b). These images are in line with those reported by Ryoo and co-workers,^20^ confirming that the material is organized in elongated tubular structures of hollow hexagonal section with 1 nm thick walls. For the mM-Z2 sample (Figure 2c,d), the hexagonal array of mesopores is not so evident (Figure 2d). At this level of resolution, however, it is not possible to distinguish, for both samples, the micropores of the MFI zeolitic system on the mesoporous walls. More detailed information about the presence of microchannels will be obtained from the textural analysis reported in the next section.

**Figure 3:**
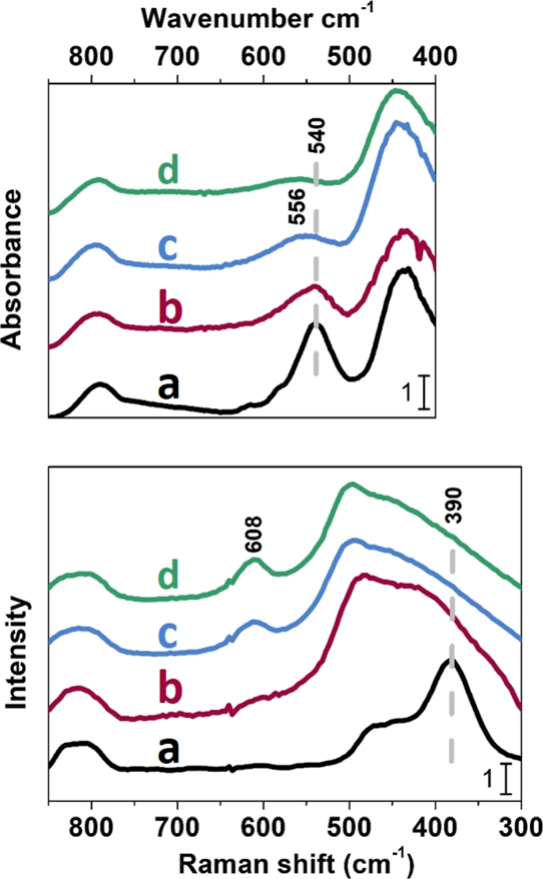
Top panel: ATR-IR spectra. Bottom panel: Raman spectra of reference ZSM-5 (a), mM-Z1 (b), mM-Z2 (c), and Al-MCM-41 (d) in the spectral region corresponding to the framework Si–O vibrational modes. Spectra have been normalized to the Si–O stretching overtone modes and shifted on the *Y* axis for the safety of clarity.

As reported by Ryoo et al.,^20^ the peculiar XRD pattern is not sufficient for the precise determination of the microporous framework structure. For this reason, IR and Raman spectra have been employed to really identify the structural fingerprints of the MFI lattice of mM-Z samples. Figure 3 shows the framework vibrational mode region of ATR-IR (Figure 3 top panel) and Raman (Figure 3 bottom panel) spectra. As far as regarding the IR spectrum, three maxima are visible in the 800–400 cm^–1^ spectral region of Figure 3 for the reference ZSM-5 (a). The bands located at 790 and 435 cm^–1^ correspond to the symmetric stretching and out of plane rocking vibrations of Si–O–Si units, respectively. The signal at 540 cm^–1^ is considered the spectroscopic signature of zeolites with MFI topology, being the collective vibrational mode of condensed five-membered units of Si atoms (pentasil units).^37−41^ The band associated with pentasil framework vibrations (highlighted by the vertical dashed line) falls at 540 cm^–1^ in the mM-Z1 spectrum (b) and at 556 cm^–1^ in the mM-Z2 spectrum (c), whereas it is almost absent in Al-MCM-41 (d). Theoretical molecular dynamics simulations demonstrated that, upon decreasing the number of pentasil units, which participate in this framework mode, the band undergoes a hypsochromic shift until reaching a frequency of 650 cm^–1^, the vibration of the isolated pentasil unit.^41^ The shift to a higher frequency of this band attested in the mM-Z sample spectra suggests the materials contain a decreased number of pentasil units compared to the standard MFI framework. This strongly supports the hypothesis that the mesopore walls of the mM-Z samples are composed of a thin layer of zeolitic micropores, less than a single-unit-cell dimension of a standard MFI zeolite. The spectral behavior of mM-Z1 is closer to that of the bulk ZSM-5 (maximum at 540 cm^–1^), testifying its higher degree of crystallinity. Concerning the Raman spectra, the ZSM-5 reference exhibits a maximum at 800 cm^–1^ (symmetric Si–O–Si stretching, active in both IR and Raman), and a broad signal below 500 cm^–1^ corresponding to the Si–O–Al stretching modes. The intense component at 380 cm^–1^ (highlighted by the dotted line in Figure 3, bottom panel) is attributed to the stretching Raman active mode of pentasil-units typical of the MFI framework.^37^ A component related to pentasil stretching vibrations at 390 cm^–1^ is recognizable in the spectrum of the mMZ-1 sample (b), confirming the presence of a MFI structural organization. In contrast, the mM-Z2 spectrum is similar to the one of the amorphous Al-MCM-41 silica. The mM-Z2 spectrum presents also a signal at 608 cm^–1^, attributed to the amorphous silica.^37,42,43^ This spectral behavior does not exclude the existence of a MFI microporous framework in the mMZ-2 sample, as already proved by the XRD pattern and by the presence of the IR band at 540 cm^–1^ (even if less resolved compared to mMZ-1) due to pentasil units. The differences observed in the mMZ-2 sample simply point out that the crystalline MFI domains of its mesoporous walls are less extended.

### 3.2 Textural Properties

**Figure 4:**
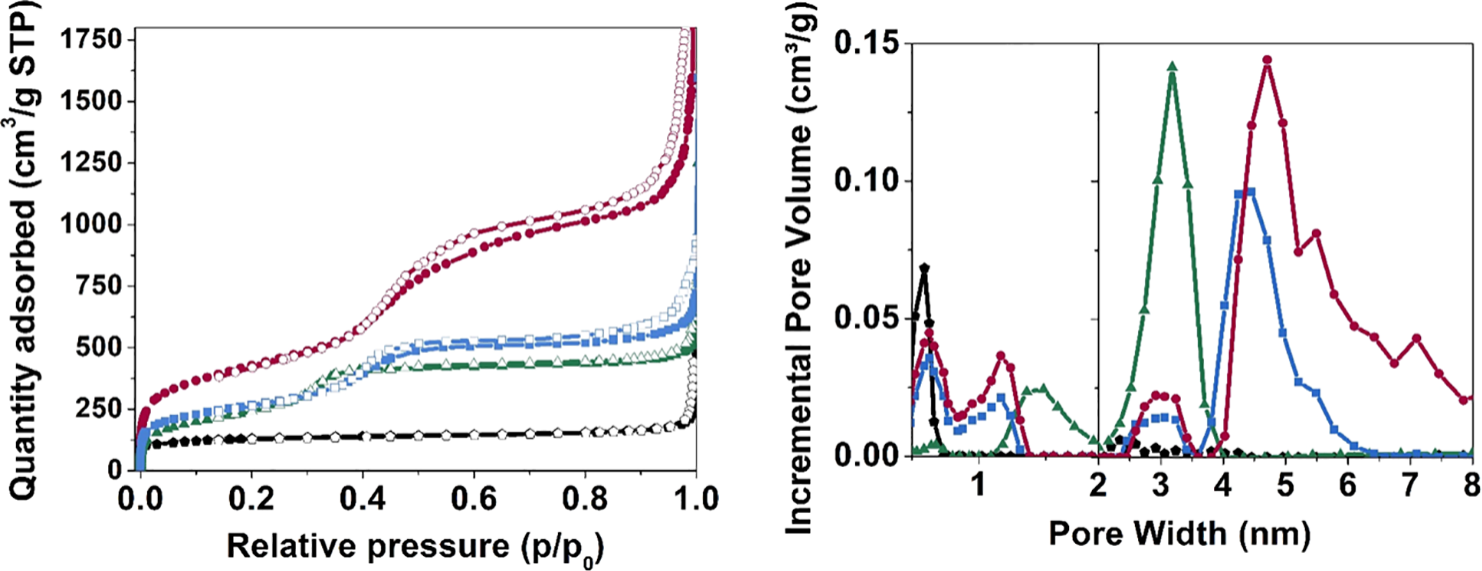
Top panel: N_2_ adsorption–desorption isotherms collected at liquid nitrogen temperature of: reference ZSM-5 (black ⬟), mM-Z1 (red ●), mM-Z2 (blue ■), and Al-MCM-41 (green ▲), filled symbols refer to adsorption and empty symbols to desorption. Bottom panel: compared pore size distribution calculated using the NL-DFT model of: reference ZSM-5 (black ⬟), mM-Z1 (red ●), mM-Z2 (blue ■), and Al-MCM-41 (green ▲).

Figure 4 (top panel) shows the N_2_ physisorption isotherms at liquid nitrogen temperature. The pore size distribution and the cumulative pore volume, calculated by modern pore modeling techniques based on NL-DFT, are reported in Figures 4 (bottom panel) and S1. Textural properties of all samples are summarized in detail in Table 1.^44^ According to IUPAC classification,^45^ the two reference ZSM-5 and Al-MCM-41 materials possess a type I (black curve) and a type IV(b) (green curve) isotherm, respectively. The reference isotherm profile agrees with what extensively reported in the literature for pure microporous and mesoporous amorphous (like MCM-41) systems.^46−48^ On the other side, both mM-Z1 and mM-Z2 samples (red and blue curves, respectively) exhibit composite isotherms, generated by the combination of type I and type IV(a) models. Both mM-Z1 and mM-Z2 isotherms present a type H4 hysteresis loop (according to IUPAC classification)^45,49^ typical of materials containing micro- and mesoporosity such as hierarchical zeolites. The symmetrical shape of the hysteresis loops suggests a gradual process of filling/evacuation of the porous system and, at the same time, it allows excluding some pore blocking or cavitation phenomena.^50^ A certain degree of order of the mesoporous structure can be, therefore, supposed for both mM-Z materials. It is worth noting that, even if the isotherms of the two hierarchical materials present the same profile, the quantity of N_2_ adsorbed on mM-Z1 is about twice in comparison with mM-Z2, being so responsible for a significantly higher specific surface area (see Table 1) in agreement with the results obtained in the paper reported by Na et al.^20^ Concerning the pore size distribution (Figure 4b), the classical ZSM-5 exhibits only a family of ultramicropores (diameter < 1 nm) with an average size of 0.6 nm in accordance with the microchannels of the MFI lattice. In contrast, the mesoporous Al-MCM-41 presents, as a major contribution to the pore volume, a family of mesopores centered at 3.2 nm and, as a minor contribution, a family of supermicropores of 1.5 nm width, with no presence of ultramicropores. The size of the mesopores of the reference Al-MCM-41 system is also responsible for the absence of any hysteresis loop in the isotherm (green curve in Figure 4, top). Indeed, for N_2_ adsorption in cylindrical pores at liquid nitrogen temperature, hysteresis occurs for pores wider than 4 nm and, hence, for adsorbents having mesopores of smaller width, completely reversible type IV(b) isotherms are observed. Interestingly, the mM-Z samples replicate at the same time the textural features of the two reference materials. The pore size distribution of mM-Z materials (Figure 4, bottom) displays a family of ultramicropores (0.6 nm), compatible with the MFI microchannels and a family of supermicropores (1.1 nm), already attested for MCM-41.^51^ The population of mesopores in the two samples is more heterogenous if compared to the reference mesoporous sample: a contribution of around 3.0 nm (the size of the MCM-41 mesopores) is always present, in the two hierarchical materials together with a maximum between 4 and 5 nm. These cavities are compatible with the hexagonal openings recognizable in the TEM micrographs, more evident for mM-Z1 (Figure 2b) and only slightly visible in mM-Z2 (Figure 2d). The mM-Z1 sample presents a consistent increase of the total pore volume if compared to the ZSM-5 and MCM-41 references, reaching 2.04 cm^3^/g. A relevant contribution to the total pore volume is ascribable to the microporous cavities (0.4 cm^3^/g), significantly exceeding the value assessed for the bulk zeolite (0.19 cm^3^/g), whereas the difference arises from its widely extended mesoporous system (1.64 cm^3^/g). The mesopore volume of mM-Z2 (0.67 cm^3^/g) is lower compared to that of mM-Z1 and more similar to the value of the reference mesoporous Al-MCM-41 material. The mM-Z2 total pore volume overcomes the one of the mesoporous references thanks to the contribution of 0.27 cm^3^/g deriving from a family of ultramicropores, exactly of the MFI size (0.6 nm), totally absent in the amorphous Al-MCM-41. These results are particularly helpful in describing the interconnected porous structure of the mM-Z materials, showing what was not completely confirmed by the structural characterization: the dual C_18_–N_3_–C_18_ templating agent selectively acts producing the microporous system, organizing the general layout in an ordered mesostructure. In summary, it is undeniable that the textural data can be traced back to a hierarchical framework (according to the previous work reported in the literature),^49,50^ also for the less-crystalline mM-Z2.

**Table 1:** Textural Properties of Hierarchical mM-Z Samples Compared with Reference Mesoporous Al-MCM-41 and Microporous ZSM-5 Materials

sample name	BET SSA (m^2^/g)	Langmuir SSA (m^2^/g)	total pore volume^a^ (cm^3^/g)	micropore volume^a^ (cm^3^/g)	mesopore volume^b^ (cm^3^/g)	ultra-micropore size^c^ (nm)	supermicropore size^c^ (nm)	main mesopore size^c^ (nm)
reference ZSM-5	381	507	0.19	0.19	0	0.6		
mM-Z1	1544	2128	2.04	0.4	1.64	0.6	1.1	3.0–4.7
mM-Z2	962	1323	0.94	0.27	0.67	0.6	1.1	3.0–4.5
Al-MCM-41	890	1243	0.76	0.13	0.63		1.5	3.2

a Calculated using the cumulative pore volume graph (Figure S1) obtained from NL-DFT analysis of the adsorption isotherm.

b Obtained using the difference between the total pore volume and micropore volume values.

c Calculated using the pore size distribution graph (Figure 4, bottom panel) obtained from NL-DFT analysis of the adsorption isotherm.

### 3.3 Nature, Abundance, and Location of Active Sites Probed by In Situ IR Spectroscopy

Among the large arsenal of characterization techniques, transmission IR spectroscopy is certainly one of the more powerful experimental tools to obtain a detailed description of the surface properties of a porous material, specifically due to the possibility to use different probe molecules in combination with an investigative technique able to monitor, with an extremely high sensitivity, the probe-surface interaction. Ideally, molecular probes for IR studies should have a small size, to monitor surfaces in all details. However, the parallel use of various probe molecules of increasing size results in a surface site speciation able to discriminate the different active sites based on their accessibility. This advanced characterization tool is particularly useful in the presence of hierarchical structures.^25^ Indeed, following the adsorption and desorption by in situ IR spectroscopy of basic molecular probes with different proton affinity (or Lewis character) and steric hindrance allows us to obtain information regarding the acidic sites strength, amount, and different locations within the porous framework.^23,52,53^

**Figure 5:**
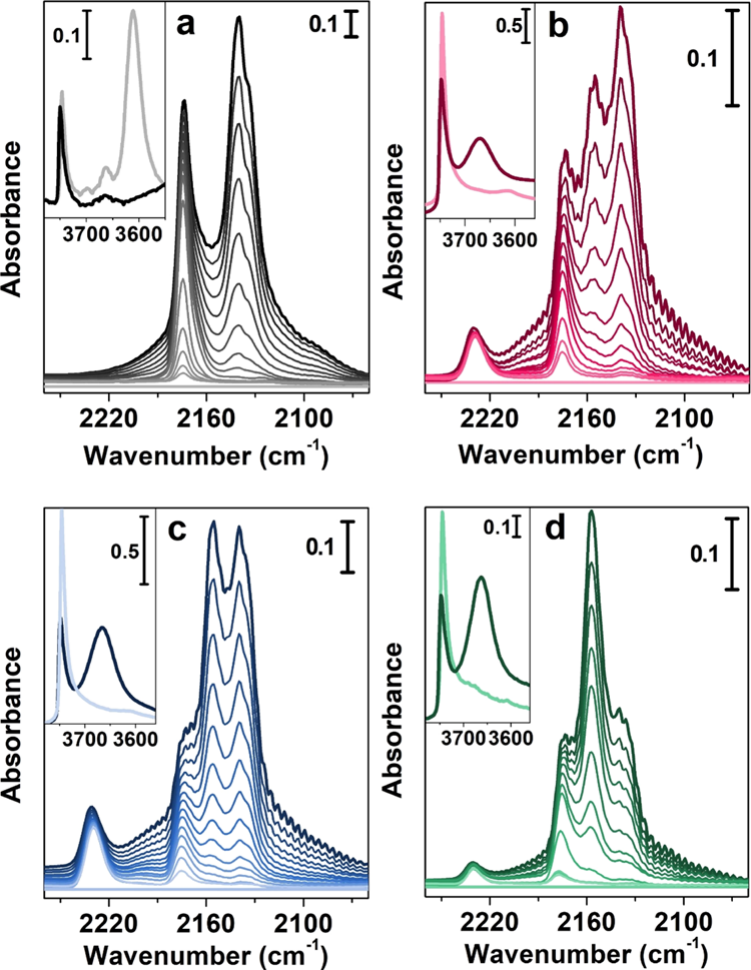
Main panels: differential IR spectra of CO adsorption at liquid nitrogen temperature in the CO stretching region (2300–2000 cm^–1^) on (a) commercial standard H-ZSM-5, (b) H-mM-Z1, (c) H-mM-Z2, and (d) H-Al-MCM-41. Strongest colors represent the highest CO coverage (80 mbar). Lighter colors indicate decreasing pressure steps. Insets: IR spectra in the O–H stretching region (3700–3500 cm^–1^) after activation in vacuum (5 × 10^–4^ mbar) at 500 °C (light curves) and after CO saturation (dark curves).

The full spectra of all dehydrated samples are reported in Figure S2 of Supporting Information, whereas the spectral region of OH stretching vibrations (ν_OH_), between 3800 and 3500 cm^–1^, is displayed in insets a, b, c, and d of Figure 5. The commercial H-ZSM-5 (Figure 5 inset a, light gray curve) spectrum is dominated by two main bands located at 3747 and 3610 cm^–1^ ascribed to isolated SiOH groups located on the external surface of the zeolite and to bridged Si(OH)Al hydroxyl groups with Brønsted acidic character, respectively.^32^ The spectra of activated H-mM-Z1, H-mM-Z2, and H-Al-MCM-41 (Figure 5 insets b, c, and d, light red, light blue, and light green curves) substantially differ from the spectrum of reference H-ZSM-5. Indeed, the OH stretching region is mainly characterized by the intense signal of virtually isolated SiOH groups at 3747 cm^–1^. For all materials, a weak band at 3610 cm^–1^ is also evident, partially overlapped to the long tail of the 3747 cm^–1^ signal, extended to lower wavenumbers, generated by terminal OH groups in hydrogen-bonded silanol chains located inside the zeolite structure (i.e., inside defective nanovoids generated by silicon vacancies).^33^ It is worth noting that the band ascribable to Brønsted bridged hydroxyl species at 3610 cm^–1^, whose intensity is proportional to the crystallization degree of the sample, is more intense in the mM-Z1 sample.

The acidic properties of the materials were first probed using CO at liquid nitrogen temperature. CO is a weak basic probe, able to detect and discriminate among acidic sites of different nature (also in the presence of rather small differences in acid strength), and, thanks to the absence of steric limitation, it can diffuse inside the zeolitic micropores. The CO adsorption/desorption spectra collected at liquid nitrogen temperature after activation at 500 °C are reported in Figure 5. As reported in the extended Figure S3, upon CO exposure, a new envelope of bands appears in the 2260–2060 cm^–1^ range and, in parallel, a clear perturbation of the OH stretching modes between 3800 and 3500 cm^–1^ occurs. Concerning the latter spectral region, a detailed explanation of the various components and of their spectral behavior is reported in the infrared spectroscopy section of the Supporting Information. The main panels of Figure 5 present the C≡O stretching region between 2260 and 2060 cm^–1^. In each figure, the spectra acquired at the highest CO coverage (the darkest curve) and upon decreasing the pressure by dynamic outgassing (lighter curves) until complete CO removal are reported. The evaluation of spectral changes at different CO coverages is extremely useful to evaluate the stability (and consequently the acid strength) of different adsorbing sites. The attribution of each band, originated by the interaction between the probe molecule and the catalyst sites, can be easily operated thanks to the extensive literature on the subject.^23,54^ In the H-ZSM-5 spectra (Figure 5a), a very intense band at 2174 cm^–1^ is visible, corresponding to the interaction of CO with the Si(OH)Al Brønsted sites. This band is very persistent also at very low CO coverages (light gray curves), indicating the strong character of these acidic sites. The signal, centered at 2140–2138 cm^–1^, is due to the liquid-like CO phase, which forms inside the zeolite micropores. The spectra of the mM-Z1, mM-Z2, and H–Al-MCM-41 samples (Figure 5b–d) exhibit the band generated by Brønsted acidity at 2175 cm^–1^, persisting until low CO coverages. The spectral behavior of this component is similar to that of the reference microporous H-ZSM-5, proving that the Brønsted Si(OH)Al acid sites of all mesoporous samples, both hierarchical (H-mM-Z1 and H-mM-Z2) and reference (H-Al-MCM-41), have possibly the same nature and the same acidic strength. Moreover, in the spectra of all mesoporous materials, a very stable band, totally absent in the reference zeolite, is visible at 2230 cm^–1^, corresponding to the interaction of CO with the polarized electronic density of Al^3+^ in the trigonal coordination, which lies in a partial framework position and possesses strong Lewis acidity.^24,32,55,56^ The Lewis acidity is basically absent in the commercial H-ZSM-5, whereas in micro–meso H-mM-Z1 and H-mM-Z2 materials, it is directly associated with an intrinsic defectivity that moves them closer to the properties of the amorphous H-Al-MCM-41. Other two components are visible in the spectra of all mesoporous materials located at 2155 cm^–1^ and at 2138 cm^–1^, ascribable to CO interacting with SiOH groups and to the liquid-like phase, respectively.

**Figure 6:**
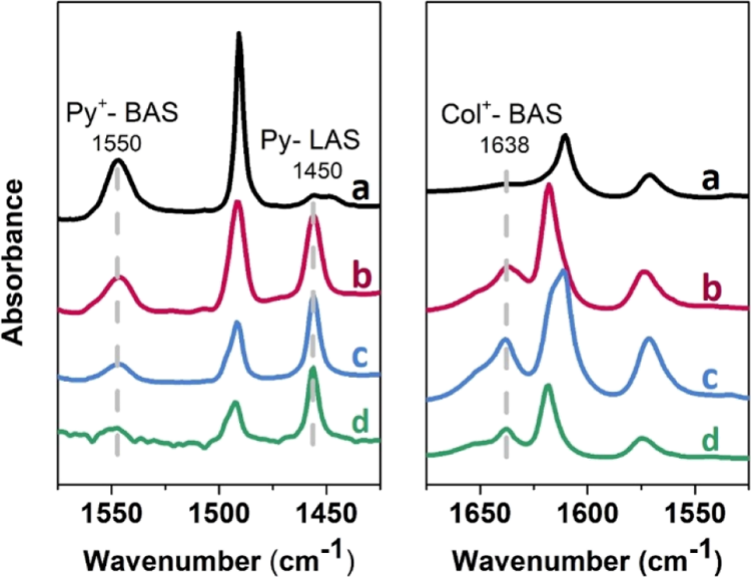
Left panel: IR spectra of pyridine adsorption on activated ZSM-5 (a), mM-Z1 (b), mM-Z2 (c), and Al-MCM-41 (d) after contact with the probe vapors at room temperature and following evacuation at 200 °C for 1 h. Right panel: IR spectra of collidine adsorption on activated ZSM-5 (a), mM-Z1 (b), mM-Z2 (c), and Al-MCM-41 (d) after contact with the probe vapor at room temperature. Spectra have been normalized to the Si–O stretching overtone modes and shifted on the *Y* axis for the safety of clarity.

After the qualitative assessment of the acidic species present in hierarchical samples by means of CO adsorption, a careful quantification of both Brønsted (BAS) and Lewis (LAS) acid sites was performed by employing pyridine (Py) as the molecular probe.^32,57^ This molecule interacts with strong Lewis acid centers and, thanks to its high proton affinity (930 kJ/mol), undergoes protonation in the presence of sites with Brønsted acid character; moreover, its kinetic diameter of 0.57 nm allows it to diffuse within the microchannels of the MFI framework (∼0.6 nm), allowing the detection of all BAS and LAS located in both micropores or mesopores. The acid site titration was performed integrating the area of the analytical 19b Py vibrational modes generated by its irreversible interaction with the acidic sites (i.e., after Py adsorption and thermal removal under vacuum at 200 °C for 1 h). IR spectra collected after sample activation and after Py contact and following outgassing at 200 °C for 2 h are reported in Figure S4 in an extended IR spectral range. The spectral region of Py ring vibrational modes is instead reported in Figure 6, left panel. The band associated with the formation of a pyridinium ion (Py adsorbed on Brønsted acid sites, Py^+^-BAS) is located at 1550 cm^–1^, whereas the band at 1450 cm^–1^ is related to the Py interaction with strong Lewis acid sites (Py-LAS). The quantitative evaluation of BAS and LAS is achievable, according to the Lambert–Beer law, by using one of the sets of experimental molar extinction coefficients available in the literature^31^ and employing the procedure reported in detail in ref (32). The results of the quantification procedure are summarized in Table 2. It should be kept in mind that this quantification method, closely related to empirical conditions, is liable to an intrinsic experimental error. This does not detract from the validity of the evaluation, if we consider it more than simply an absolute value, but a method of comparison between different samples, useful to evaluate the total acidity more in qualitative terms.

**Table 2:** Concentrations of BAS and LAS in mol/kg Calculated by In Situ IR Spectroscopy of Adsorbed Pyridine after 2 h of Outgassing at 200 °C^a^

sample name	BAS (mol/kg)	LAS (mol/kg)	total acid sites (mol/kg)
H-ZSM-5	1.099	0.149	1.248
H-mM-Z1	0.410	0.468	0.878
H-mM-Z2	0.142	0.495	0.637
H-Al-MCM-41	0.019	0.073	0.092

a The quantification has been carried out following the experimental procedure reported in ref (32) and the integrated molar extinction coefficients of ref (31).

The amount of BAS of the reference H-ZSM-5 significantly overcomes the one of the other materials, even if it contains the same concentration of aluminum atoms. The two H-mM-Z samples have a reasonable amount of BAS and at the same time, a not negligible concentration of LAS, comparable to that of H-Al-MCM-41 and ascribable to the presence of Al atoms in the trigonal coordination. The H-mM-Z1 sample, with a higher crystallinity degree, exhibits a relevant concentration of BAS, higher than that of LAS, as for the bulk H-ZSM-5. Contrarily, the less-crystalline H-mM-Z2 presents a higher concentration of LAS, almost twice the amount of BAS, with a similar trend to the reference H-Al-MCM-41.

It is worth noting the total amount of acidic sites follows the trend H-ZSM-5 > H-mM-Z1 > H-mM-Z2 > H-Al-MCM-41, exactly coincident with the crystallinity degree of the materials.

Dotted gray lines underline the vibrational band identifying the formation of the pyridinium ion (Py^+^-BAS), pyridine interaction with acidic Lewis centers (Py-LAS), and collidinium ion formation (Col^+^-BAS).

A step forward in the study of acid site speciation has been made using the sterically hindered 2,4,6-trimethyl pyridine (2,4,6-collidine, Col) as a molecular probe. The high proton affinity of this molecule (980 kJ/mol) allows the formation of collidinium ion in the presence of sites with Brønsted acid character (Col^+^-BAS) but, due to the three substituents on the pyridine ring, its kinetic diameter (0.74 nm) exceeds the size of the narrow microchannels of the MFI zeolite.^58,59^ It is thus possible to make a distinction between the acidic sites located on the outer surface of the material (which can interact with Col) and on the inner surface of the micropores, which is inaccessible to this sterically hindered probe molecule. The full spectrum of the three samples before and after contact with collidine vapors is shown in Figure S5. The interaction with Col causes a clear perturbation of the OH-stretching region (3800–3200 cm^–1^) and the appearance of new absorption bands in the ring vibrational mode range (1700–1500 cm^–1^). For what concerns H-ZSM-5, the band at 3747 cm^–1^, ascribed to the isolated SiOH group, is completely consumed, whereas the component at 3610 cm^–1^, due to bridged Si(OH)Al species, is substantially unperturbed. This is a clear indication of the location of the different OH groups. Indeed, if a silanol stretching band is affected by the adsorption of Col, it means that the species are located outside the microchannels. In parallel to the consumption of the band at 3747 cm^–1^, a very broad envelope appears below 3650 cm^–1^, produced by the H-bonding interactions of external SiOH and AlOH species with the probe molecule. In contrast, the band at 3610 cm^–1^ is still visible after Col contact (even if perturbed due to the superimposition with the broad bands produced by OH groups interacting by H-bonding with Col), indicating that the BAS are not accessible to the hindered molecule, because they are essentially located inside the MFI micropores. The region of Col ring vibrational modes is reported in Figure 6, right panel (curve a). The spectrum of Col vapors contacts with the H-ZSM-5 exhibits two main components, at 1619 and 1575 cm^–1^, ascribable to Col interacting with isolated external OH species. Another weak signal is also present at 1638 cm^–1^, suggesting that the collidinium ion forms to a very limited extent, due to the presence of a small fraction of Brønsted acid sites approachable by Col. This spectral behavior suggests these BAS are located at the micropore mouth, and so exposed to the external surface. Concerning the H-mM-Z samples (Figure 6 right panel, b and c curves), the component at 1638 cm^–1^ is more intense, suggesting that a significant amount of Col has been protonated by interaction with BAS. It means that a non-negligible amount of BAS is accessible to Col and, therefore, reasonably located at the micropore mouth. The interaction of Col with SiOH groups is well visible in both Figures S5 and 6 (right panel). The band at 3747 cm^–1^ is only partially eroded in the H-mM-Z samples, suggesting that a fraction of the silanols is not accessible to the bulky molecule. The not accessible SiOH species are associated with hydrogen-bonded silanol chains present in the defects (nanovoids generated by silicon vacancies) of the zeolitic framework and are absent in the reference H-ZSM-5. The presence of defectivity in both H-mM-Z samples reflects the difficulty of crystallization of these micro–mesostructures, with an evident perturbation of the long-range structural ordering of the zeolitic framework along the walls of the mesoporous channels. The use of size-selective probe molecules is particularly informative in the study of a hierarchical material, especially if coupled the use of a molecular probe with suitable dimensions to spread inside the micropores (namely CO at liquid nitrogen temperature).^61,62^ Indeed, the consecutive adsorption of Col and CO helps to discriminate the fraction of sites not accessible to collidine, but available to interact with a probe that can easily enter the microchannels. For this reason, adsorption of Col vapors was preliminary carried out on the activated H-mM-Z2 sample, saturating all the accessible sites, then the adsorption/desorption of CO at liquid nitrogen temperature was performed according to the above-reported procedure (Figure S6). A comparison with Figure 6 (right panel) clearly highlights that the band at 2230 cm^–1^ (CO interacting with trigonal Al^3+^ species) is no more present, while the component at 2157 cm^–1^ (CO interacting with Si–OH groups) persists. The band ascribable to Si(OH)Al Brønsted acidic species at 2177 cm^–1^ is instead very weak and distinguishable at very low CO coverages. Summarizing, in hierarchical H-mM-Z materials, trigonal Al^3+^ species with Lewis acid character are not located inside the micropores, whereas just a small fraction of BAS is unavailable for collidine adsorption, indicating that these sites are almost completely concentrated in close proximity to the micropore mouths. This arrangement of the Brønsted acidic species further confirms the walls of the mesoporous channels are made of thin layers of the MFI framework, in which the periodicity of the zeolite is retained in one dimension.

### 3.4 Catalytic Hydroconversion of *n*-Decane

The analysis of the products of the hydroconversion reaction of a model long-chain *n*-alkane gives precious information about the location of active sites and about the inner space architecture of a zeolitic structure. To this purpose, the acidic zeolite was loaded with Pt particles (as reported in the Experimental Section) constituting a bifunctional catalyst. The catalytic hydroconversion involves two different types of active sites, the noble metal particles and the Brønsted acid. The reaction is initiated by the dehydrogenation of the alkane by the action of the noble metal functionality to form an alkene. The acidic Brønsted site can protonate the alkene forming an alkylcarbenium ion. The rearrangement of this cationic intermediate drives the generation of structural isomers passing by a cyclic iso-alkylcarbenium ion intermediate and then the cracked products can be generated by β-scission.^27^ The shape selectivity of a zeolite drives the location of the positive charge along the linear chain and thus the positional selectivity of chain branching or breaking.^63^ The steric limitations imposed by a zeolitic lattice, combined with the distribution of the active sites, stabilizes specific conformation of the alkylcarbenium ion intermediates, thus reflecting in the selectivity for specific isomers and cracked products. A list of specific criteria and the tabulation according to the opening diameter of the channels of the most common zeolite frameworks makes the “*n*-decane test” an easy-to-read tool for disclosing new structures and their modifications.^64−66^ It was applied here to investigate the activity as well as the spatial constraints around the active sites contributing to the catalytic activity.

**Figure 7:**
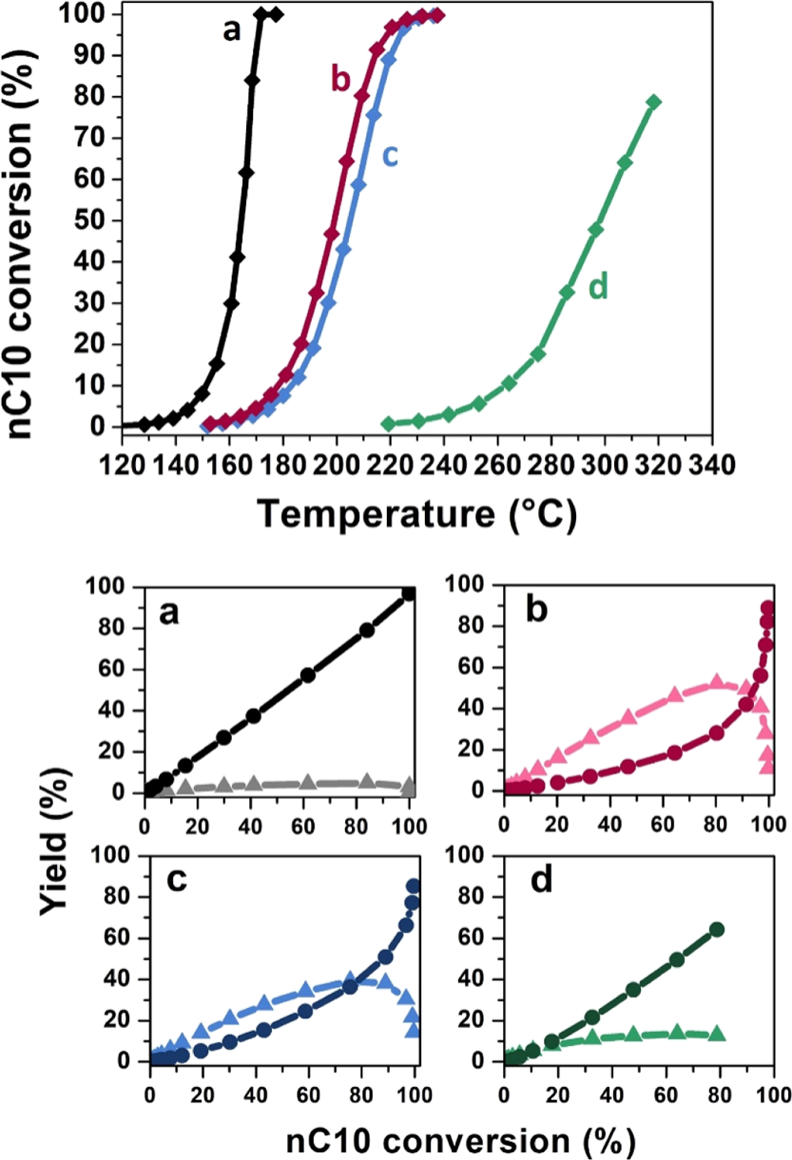
Top panel: Conversion curves of *n*-decane over commercial H-ZSM-5 (a), H-mM-Z1 (b), H-mM-Z2 (c), and H-Al-MCM-41 (d): the percentage of *n*-decane converted moles is reported as a function of increasing reaction temperature. Bottom panel: yields of skeletal isomers (light lines) and cracked products (dark lines) as a function of *n*-decane converted by commercial H-ZSM-5 (a), H-mM-Z1 (b), H-mM-Z2 (c), and H-Al-MCM-41 (d).

The *n*-decane conversion curves, as a function of the reaction temperature, are reported in Figure 7 (top panel) for all the samples. The *n*-decane conversion is complete at 170 °C over the reference H-ZSM-5 catalyst (curve a), whereas the hierarchical H-mM-Z samples need 230 °C (curves b and c). In contrast, the amorphous H-Al-MCM-41, even above 300 °C, is still not able to completely convert the feed hydrocarbon (curve d). The apparent activation energy values, computed from Arrhenius plots reported in Figure S7, exactly reflect the large differences between the samples in terms of activity (Table 3). The significantly higher activity of the commercial H-ZSM-5 sample must be attributed to the much higher concentration of active acid sites (as already discussed in the section dedicated to the IR study of pyridine adsorption) guaranteed by the bulk zeolitic structure, where the Brønsted acidic sites are located inside the extended microporous lattice. On the contrary, the hierarchical H-mM-Z samples present a lower concentration of BAS, compensating by a high amount of LAS and a high concentration of isolated and terminal silanols. The lower activity of these two samples is also related to the short extension of the crystalline MFI domains. The adsorption/diffusion processes, being part of the total catalytic process, are strongly influenced by the rigidity of the framework and by its extension. The more flexible (i.e., amorphous) a structure is, the less active it appears, because the adsorption/diffusion of the feed molecule is hindered. The same happens when the dimension of the crystals decreases. This consideration gains importance as the amorphous degree increases, as for the H-mM-Z2 sample compared to H-mM-Z1 and mostly to the bulk H-ZSM-5.

**Table 3:** List of the Main Products and Principal Parameters of the *n*-Decane Test

	H-ZSM-5	H-mM-Z1	H-mM-Z2	H-Al-MCM-41
apparent activation energy (kJ/mol)^a^	179	150	144	128
refined constraint index (CI°)^b^	4.66	3.25	4.03	1.66
ethyloctane (%) in monobranched isomers at 5% isomerization yield	3.16	0.21	0.18	9.57
propylheptane (%) in monobranched isomers at 5% isomerization yield	0.0	0.0	0.0	0.0
dibranched isomers (%) at maximum isomerization yield	21.05	14.58	17.58	12.48
amount of 2,7-DMC8 (%) in dibranched isodecanes at 5% of dibranching yield	21.21	15.50	18.90	5.77
C3–C7 (mol/100 mol C10 cracked) at 35% cracking yield	12.21	7.56	5.68	11.86
C4–C6 (mol/100 mol C10 cracked) at 35% cracking yield	6.11	3.70	2.50	4.44
C5 isomers (mol/100 mol cracked) at 35% cracking yield	11.82	21.33	21.51	1.36
dimensionality index (DI°)^c^	18.32	11.26	8.18	16.30

a Calculated using the Arrhenius equation ln *k* = −*E*_a_/*RT* + ln *A* from the plots reported in Figure S7.

b Ratio of the yield of 2-methylnonane to 5-methylnonane at 5% isomerization yield.

c Sum of the fractions (|C3–C7| + |C4–C6|) at 35% of cracking yield.

Figure 7 (bottom panel) shows the yield of products of *n*-decane hydroconversion over the different catalysts, divided over skeletal isomers and cracked products. The 10 membered ring (10MR) H-ZSM-5, with a limited internal space, prevents the formation of structural isomers and promotes the formation of shorter cracked products. This catalytic behavior is visible in Figure 7 (bottom panel, section a), where the cracking yield (dark line) grows linearly starting from low conversion values, while the isomerization yield (light curve) is basically close to zero. In contrast, the H-mM-Z samples exhibit an opposite trend as reported in Figure 7 (bottom panel, sections b and c): the isomerization yields (light red and light blue curves) override the darkest curves due to cracking from the very early stages of the reaction and only when the conversion overcomes 75%, an inverse trend is observed. If the isomerization yield of reference H-ZSM-5 does not exceed 10%, it reaches 50 and 40% for H-mM-Z1 and H-mM-Z2, respectively. This behavior can be explained by the presence of mesopores, which allows the stabilization of cyclic transition states that lead to the isomerization of the chains. At the same time, thanks to mesoporous cavities, the branched chains can be easily expelled from the hierarchical framework as structural isomers, having a higher steric hindrance than linear substrate chains or cracked fractions. At high conversions, cracking products (a mixture of linear and branched molecules) prevail. The reference H-Al-MCM-41 sample has a totally different catalytic trend compared to the hierarchical materials, with a prevalence of linear cracked products. The occurrence of hydrogenolysis instead of hydrocracking, driven by the lower amount of Brønsted acid sites of the purely mesoporous samples, could explain the different product distribution of this material.

In 10-membered ring zeolites such as MFI, shape selectivity influences the composition of skeletal isomers from *n*-decane.^67,68^ The distribution of main products is summarized in Table 3 and Figure S8 and described in detail in the Supporting Information. The selectivity of the catalysts is directly expressed by the calculated constraint index^66^ CI° values, which are 3.25 for H-mM-Z1, 4.03 for H-mM-Z2, 4.66 for the commercial H-ZSM-5, and only 1.66 for H-Al-MCM-41. A CI° value close to 1 suggests the absence of shape selectivity (usually for the MFI, the CI° exceeds a value of 2.7). In contrast, the prevalence of isomerization to form 2-methyl-nonane proves the existence of a 10MR structure in the H-mM-Z samples and the PCP transition-state shape selectivity typical of the MFI topology. Another proof testifying the existence of the MFI framework in the H-mM-Z samples is the prevalence of the 2,7-methyloctane di-branched product. The branching occurring at the extremities of the chain is always induced by an acidic site located at the pore mouth, the only point within the MFI lattice, where there is enough space for hosting the cyclopropane intermediate. In this specific case, the isomerization is induced by the action of two sites lying in the mouth of very close pore openings, where the substrate chain can be positioned only in a key-lock configuration. The percentage of 2,7-dimethyloctane produced is reported in Table 3. From these results, it is possible to conclude that the H-mM-Z samples are shape selective and active thanks to acidic sites mainly located at the mouth of the MFI microchannels (as already proved by the in situ IR study of adsorbed collidine), working as the acidic sites of a bulk H-ZSM-5.

**Figure 8:**
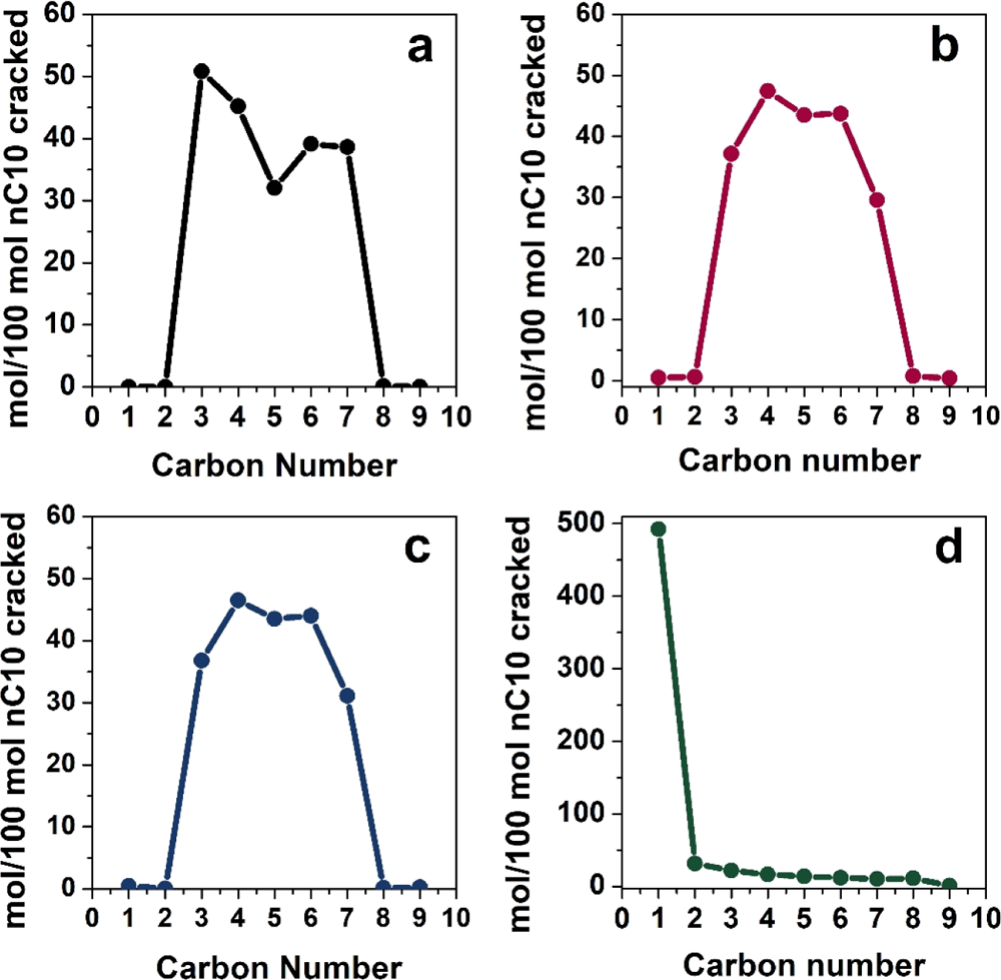
Distribution of *n*-decane cracked products per carbon number chain length, in the presence of commercial H-ZSM-5 (a), H-mM-Z1 (b), H-mM-Z2 (c), and H-AlMCM-41 (d). Products obtained at ca. 35% *n*-decane hydrocracking conversion.

The analysis of individual cracked product fractions (grouped by the carbon atom number) offers other relevant information about the microporosity of the materials (Figure 8). The bifunctional mechanism of hydrocracking of *n*-alkanes involves a preferential β-scission of the alkylcarbenium ion intermediate, consecutively to branching steps. Short chains are preferentially produced by the narrow MFI framework, while if no geometrical restrictions exist (the zeolite Y case), the splitting occurs in the central position, giving rise mainly to the C5 chains. For ZSM-5, the distribution of cracked products displays a typical “M-shape”, with a minimum for C5 products and two maxima positioned at C3–C7 or C4–C6 (depending on the specific sample), due to the preferential branching and cracking at the extremities of the C10 chains. A symmetrical “M shape” testifies the prevalence of primary cracking. Secondary cracking usually occurs in systems with narrow channels as MFI, whence the fragments escape after a long residence time. The sum of |C3–C7| + |C4–C6| molar yields defines the dimensionality index (DI°). The lower this index, the more symmetrical the “M curve” and the more primary cracking is favored. As reported in Figure 8, the reference H-ZSM-5 and both H-mM-Z samples exhibit a “M”-shaped curve, the fingerprint of the MFI topology. The cracked product distribution of H-ZSM-5 (Figure 8a) presents an absolute maximum for the C3 fragments, the shortest molecules that can be obtained via β-scission. Indeed, its high DI° value of 18.32 reveals the cracking is occurring in a constrained molecular environment.

On the other hand, H-mM-Z samples (Figure 8b,c) have a more symmetrical “M curve” (and lower DI° index), with a content of the C5 fraction higher than 40% and two maxima in correspondence of C4–C6 fractions. This trend suggests that cracking of the C10 chains occurs with less geometrical restrictions, due to the increased product diffusion favored by the mesoporous channels. This fact limits the secondary cracking and allows C10 skeletal isomers to be released before cracking. In contrast, H-Al-MCM-41 reference does not display any shape selectivity, with a complete conversion of the decane to methane (Figure 8d), reasonably produced by hydrogenolysis on Pt particles. If only primary cracking occurs, the total sum of the molar yields of each fraction of cracked products out of 100 mol of feed *n*-decane should sum up to 200 mol per 100 mol *n*-decane cracked. A deviation from this marker number stands for secondary cracking reactions. Figure S9 shows the trends for the different catalysts. Again, the results prove how the micropores exposing the acid sites and the large space offered by the mesoporous channels in H-mM-Z materials work together to achieve performances resembling a standard H-ZSM-5, but at the same time differing in terms of diffusivity and mass transport.

## 4 Conclusions

Two hierarchical structures were synthesized following the procedure reported by Ryoo and co-workers,^20^ employing the dual-porogenic surfactant C_18_–N_3_–C_18_ as the templating agent. After the replication and optimization of the synthetic strategy, an advanced and unique characterization approach was carried out, combining spectroscopic tools and targeted catalytic tests. These characterization techniques were the toolbox for disclosing the amount, location, and distribution of the active sites, in order to really understand how the multilevel porosity affects the catalytic activity and the shape-size selectivity.

By reproducing the synthesis procedure reported in the literature, it was found that the gel temperature during the aging phase considerably affects the final characteristics of the materials and, mainly, the level of crystallinity. Both materials possess an ordered mesostructure containing an ultramicroporous network exactly comparable in size to the microcavities of a standard ZSM-5; however, the extent of the microporous MFI domains is lower for the mM-Z2 sample. The acid sites were investigated by means of in situ IR spectroscopy with different molecular probes. The adsorption of CO proved the nature and the acid strength of the Brønsted sites were equivalent to those of a standard H-ZSM-5, whereas their concentration (quantified by pyridine adsorption) was significantly lower. In contrast to the standard zeolite, acidic sites with Lewis character were also detected in both hierarchical materials due to their intrinsic defectivity (the presence of Al^3+^ in partial framework positions), comparable to the reference Al-MCM-41. The final proof of the existence of a microporous MFI framework, really integrated with the ordered mesostructure, was obtained by the *n*-decane test.^69^ The catalytic results proved the hierarchical samples exhibited a shape selectivity of a 10-membered ring lattice and the distribution of *n*-decane conversion products typical of a H-ZSM-5, operated by the action of Brønsted acid sites located at the mouth of the micropores. In addition, the transport and diffusion properties of products of micro–/meso- materials were superior to those of a standard bulk zeolite, due to the presence of the mesoporous network, which limits the secondary cracking and, at the same time, allows an easy release of the structural isomers. The evaluation of intermediates and products of the *n*-decane test unveiled the totally new shape-size selectivity determined by the interconnectivity of micropores and mesopores.

Surprisingly, these catalytic features were even more pronounced in the H-mM-Z2 sample than in H-mM-Z1, which instead possesses more extended MFI crystal domains. However, even if H-mM-Z2 is more similar to a standard ordered mesoporous silica, its catalytic behavior cannot be questioned, as highlighted by the total absence of activity and selectivity of the mesoporous H-Al-MCM-41 reference.

By merging the data of this advanced multitechnique characterization approach and comparing the results of the catalytic *n*-decane hydroconversion, performed on other hierarchical structures (such as two-dimensional MFI nanosheets),^70^ we proved the hierarchical mM-Z samples are constituted by mesoporous channels with a hexagonal array, whose walls are made up of a thin layer of MFI domains, displaying almost all acidic sites at the micropore mouths, along the mesochannels’ internal surface (totally accessible to bulky substrates, as testified by the easy adsorption of collidine).

For all these reasons, the structure of mM-Z can be effectively categorized in the definition of “reverse hierarchy” (a porous system, where a collection of small pores flows together into a larger pore to gain a faster outlet).^49^ Finally, it is worth noting that the presence of more extended crystalline MFI domains in the mesopores’ walls does not improve the catalytic performances in *n*-decane hydroconversion, but the synergy between the two different levels of porosity is responsible for the peculiar catalytic activity and shape selectivity of these hierarchical zeolites.

## Supporting Information Available

N_2_ physisorption at liquid nitrogen temperature (cumulative pore volume distribution calculated using the NL-DFT model); transmission IR spectroscopy (IR spectra of the activated samples, IR spectra of the activated samples upon contact with CO, IR spectra of the activated samples upon contact with pyridine, IR spectra of the activated samples upon contact with collidine, and IR spectra of the activated samples upon contact with collidine and then with CO); *n*-decane hydroconversion (Arrhenius plots, *n*-decane mono-branched isomers, and total amount of *n*-decane fractions per 100 mol of *n*-decane cracked) (PDF)
