# Supplementary material for: Insights on a Hierarchical MFI Zeolite: A Combined Spectroscopic and Catalytic Approach for Exploring the Multilevel Porous System Down to the Active Sites
Source: ACS Appl Mater Interfaces. 2021 Sep 20;13(41):49114–27. doi: 10.1021/acsami.1c11614 (PMC8532120; doi:10.1021/acsami.1c11614)
Supplement: Supplementary file 1 — am1c11614_si_001.pdf [file am1c11614_si_001.pdf]

Supporting Information (SI)

Insight on a hierarchical MFI zeolite: a combined spectroscopic and catalytic approach for exploring the multilevel porous system down to the active sites

Alessia Airi,[1] Matteo Signorile,[1] Francesca Bonino,[1] Pierluigi Quagliotto,[1] Silvia Bordiga,[1] Johan A. Martens,[2] Valentina Crocellà[1]*

[1] Department of Chemistry, NIS and INSTM reference centre, University of Turin, via G.Quarello 15/A 10135 and via P.Giuria 7 10125, Turin, Italy

[2] KU Leuven, Centre for Surface Chemistry and Catalysis, Celestijnenlaan 200F, Box 2461, B-3001 Leuven, Belgium

*Corresponding author: valentina.crocella@unito.it

Table of content:

- ***N_2 physisorption at liquid nitrogen temperature***

Figure S1: cumulative pore volume distribution calculated by NL-DFT model...2

- ***Transmission IR spectroscopy***

Figure S2: IR spectra of activated samples...3

Figure S3: IR spectra of activated samples upon contact with CO..3

Figure S4: IR spectra of activated samples upon contact with Pyridine..4

Figure S5: IR spectra of activated samples upon contact with Collidine...5

Figure S6: IR spectra of activated samples upon contact with Collidine and then with CO.............................5

- ***n-decane Hydroconversion***

Figure S7: Arrhenius plots...6

Figure S8: n-decane mono-branched isomers...7-8

Figure S9: total amount of n-decane fractions per 100 moles of n-decane cracked..................................9

- ***N_2 physisorption at liquid nitrogen temperature***

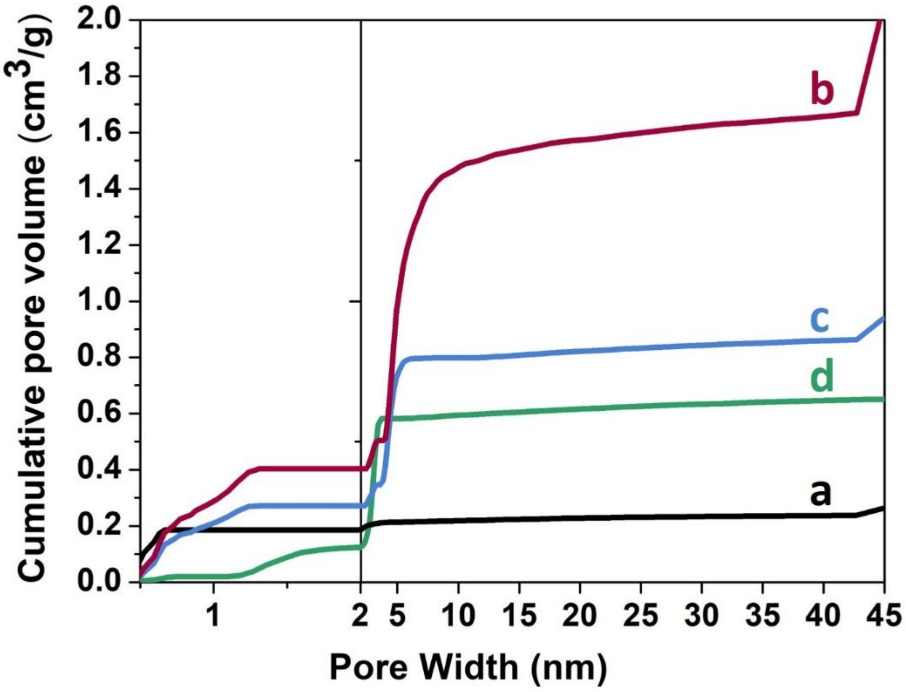

***Figure S 1**. Compared cumulative pore volume distribution calculated by NL-DFT model of: reference ZSM-5 (a) mM-Z1 (b), mM-Z2 (c), Al-MCM-41 (d).*

- ***Transmission IR spectroscopy***

IR spectra of activated samples

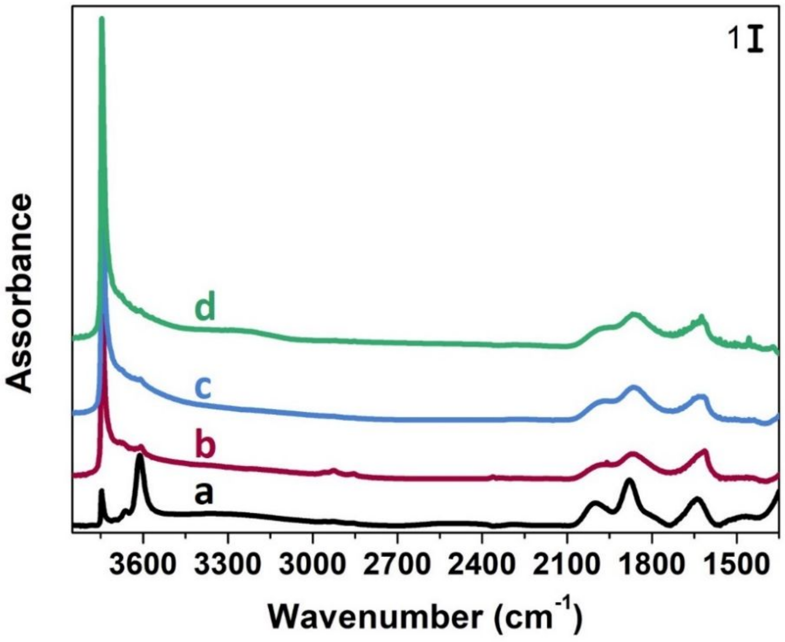

Figure S 2. *IR spectra, in the 3800-1350 cm^{-1} range of activated samples: H-ZSM-5 (a), H-mM-Z1 (b), H-mM-Z2 (c) and H-Al-MCM-41 (d). Spectra have been normalized to the Si-O stretching overtone modes and shifted on the Y axis for the safe of clarity.*

IR spectra of activated samples upon contact with CO

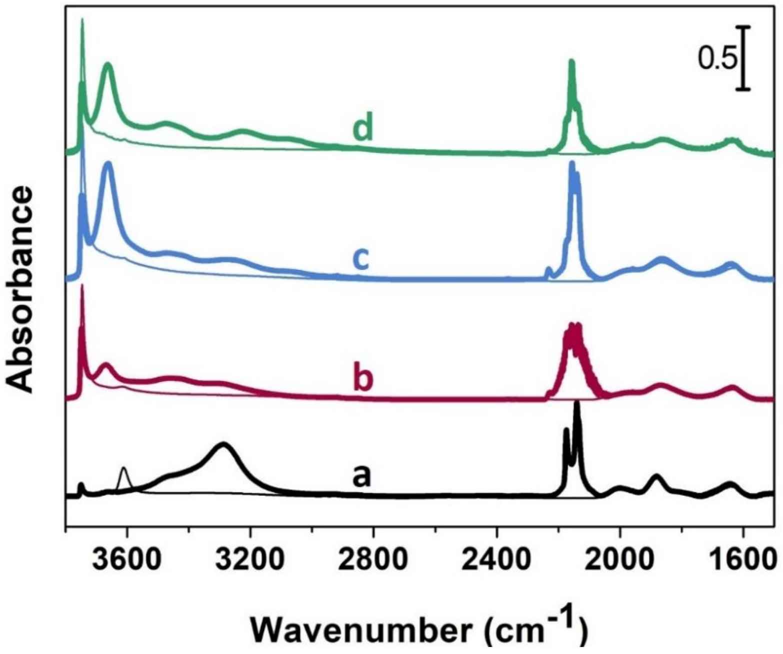

Figure S 3*. IR spectra, in the 3800-1500 cm^{-1} range of activated samples (thin lines) and in presence of 80 mbar CO equilibrium pressure (bold lines): H-ZSM-5 (a), H-mM-Z1 (b), H-mM-Z2 (c) and H-Al-MCM-41 (d). Spectra have been normalized to the Si-O stretching overtone modes and shifted on the Y axis for the safe of clarity*

Figure S3 displays the perturbation induced by the adsorption of CO in the spectra of activated materials (thin versus bold curves). Upon exposure to the highest CO coverage (bold curves, 80 mbar of CO equilibrium pressure), a new envelope of bands appears in the C≡O stretching region between 2260 cm^{-1} and 2060 cm^{-1} and, in parallel, a clear perturbation of the OH stretching spectral region between 3800 cm^{-1} and 3500 cm^{-1} occurs. The detail of the two spectral region is reported in Figure 5 of the main text (in both insets and main panels). Concerning the OH spectral region, the H-ZSM-5 reference sample (Figure 5 - inset a) the band of virtually isolated SiOH groups (3747 cm^{-1}), is only slightly perturbed, while a weak, broad and not resolved signal at around 3650 cm^{-1} appears, indicating a definitely poor interaction of these species with the probe molecule, as already reported for zeolites presenting a very high concentration of Brønsted acidic sites.[1]At the same time, the band at 3610 cm^{-1}, related to the Brønsted Si(OH)Al acid site, is completely eroded. As reported in the extended figure S3, the above mentioned component shifts to 3300 cm^{-1} due to the interaction with CO, as commonly found for a H-ZSM-5.[2,3] The behaviour of H-mM-Z1, H-mM-Z2 and H-Al-MCM-41 again differs from the reference H-ZSM-5. As reported in the insets b, c and d of Figure 5 (dark curves), the band at 3747 cm^{-1}, related to isolated SiOH species, is partially eroded and shifted to 3665 cm^{-1} for all the mesoporous samples.

IR spectra of activated samples upon contact with Pyridine

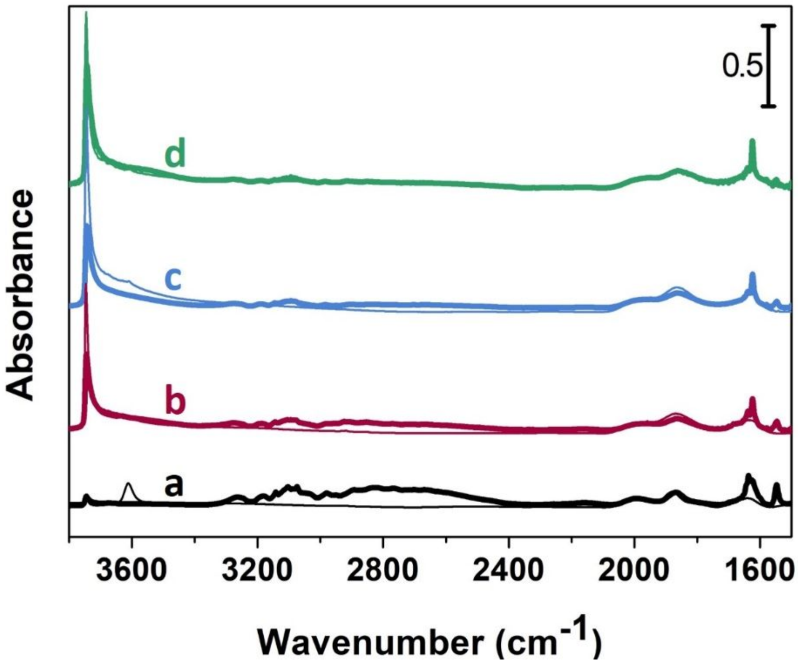

***Figure S 4**. IR spectra, in the 3800-1500 cm^{-1} range, of activated samples (thin lines) and in presence of pyridine vapour pressure (bold lines): H-ZSM-5 (a), H-mM-Z1 (b), H-mM-Z2 (c) H-Al-MCM-41 (d). Spectra have been normalized to the Si-O stretching overtone modes and shifted on the Y axis for the safe of clarity.*

IR spectra of activated samples upon contact with Collidine

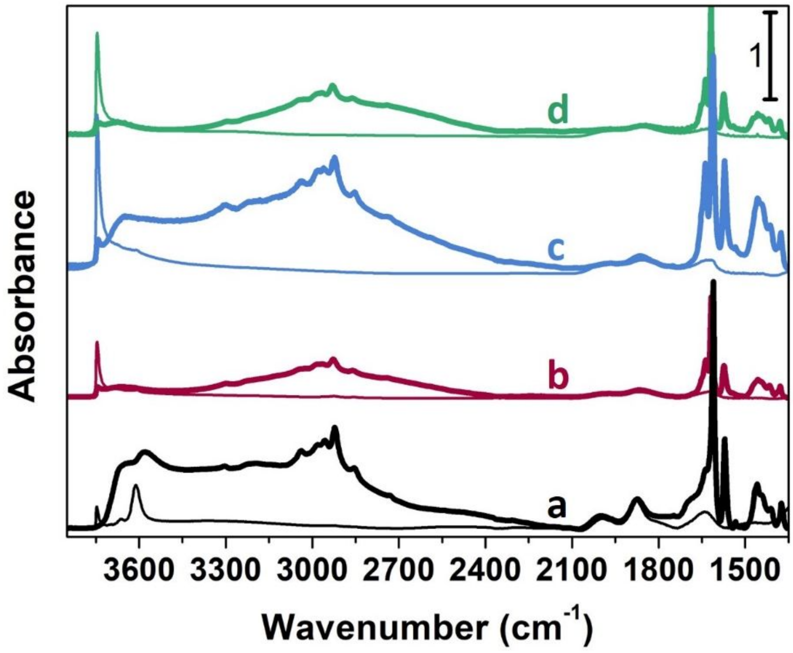

***Figure S 5**. IR spectra, in the 3800-1500 cm^{-1} range of activated samples (thin lines) and in presence of collidine vapour pressure (bold lines): H-ZSM-5 (a), H-mM-Z1 (b), H-mM-Z2 (c) and H-Al-MCM-41 (d). Spectra have been normalized to the Si-O stretching overtone modes and shifted on the Y axis for the safe of clarity*

IR spectra of activated samples upon contact with Collidine and then with CO

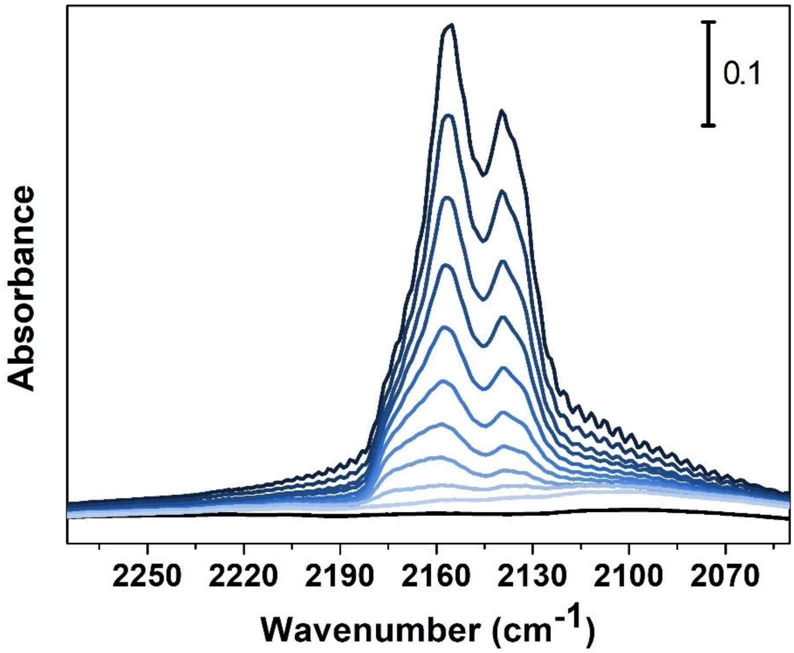

***Figure S 6**. IR spectra in the CO stretching region of activated H-mM-Z2 after saturation with collidine vapours and consecutive adsorption of CO at 77 K. Before CO adsorption, physisorbed collidine was removed through a long degassing at room temperature.*

- ***n-decane Hydroconversion***

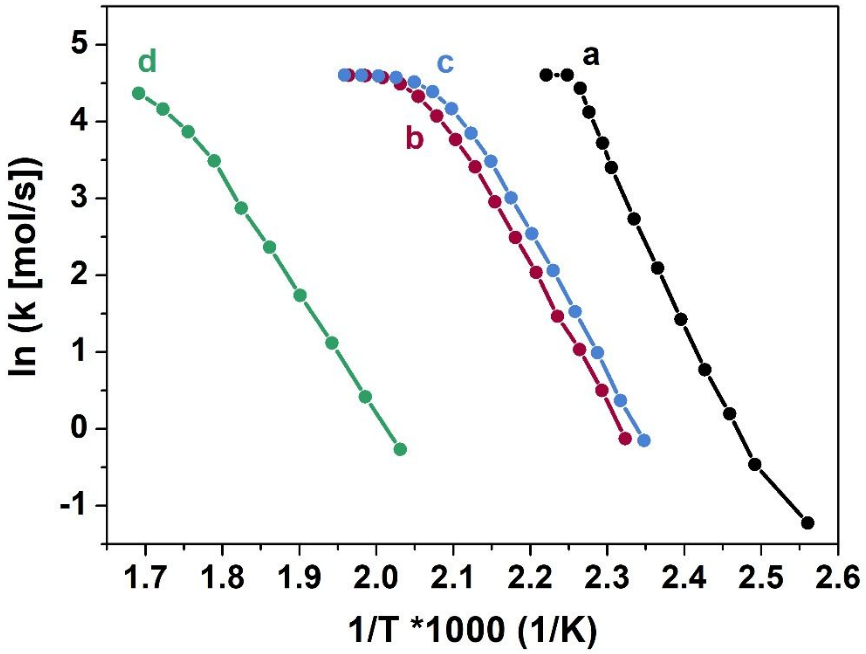

Figure S 7. Dependence of reaction rate of n-decane conversion on temperature in the form of Arrhenius plots for reference H-ZSM-5 (a) H-mM-Z1(b), H-mM-Z2 (c), H-Al-MCM-41 (d).

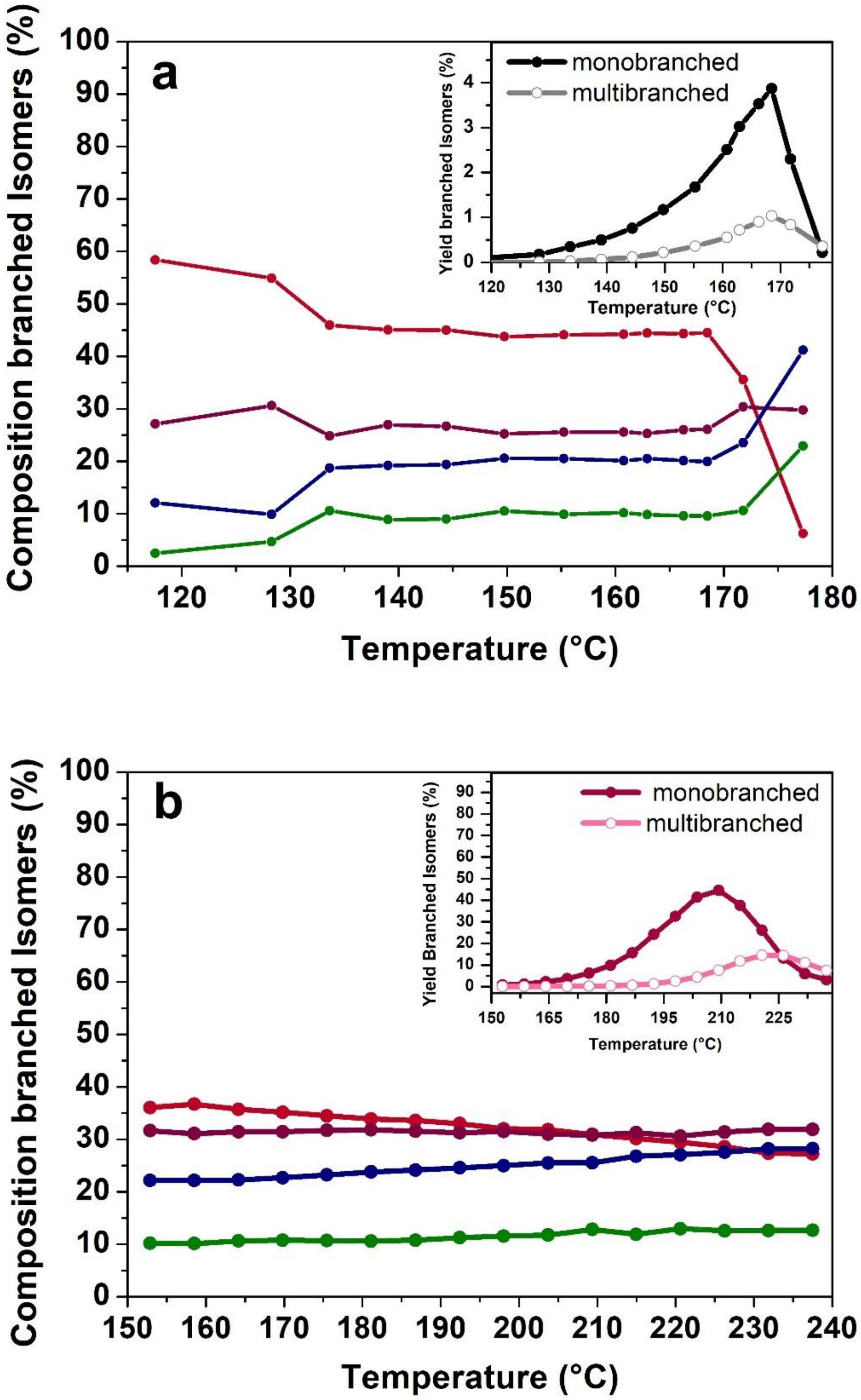

a
Composition branched Isomers (%)
Temperature (°C)
monobranched
multibranched
Yield branched Isomers (%)
Temperature (°C)
b
Composition branched Isomers (%)
Temperature (°C)
monobranched
multibranched
Yield Branched Isomers (%)
Temperature (°C)

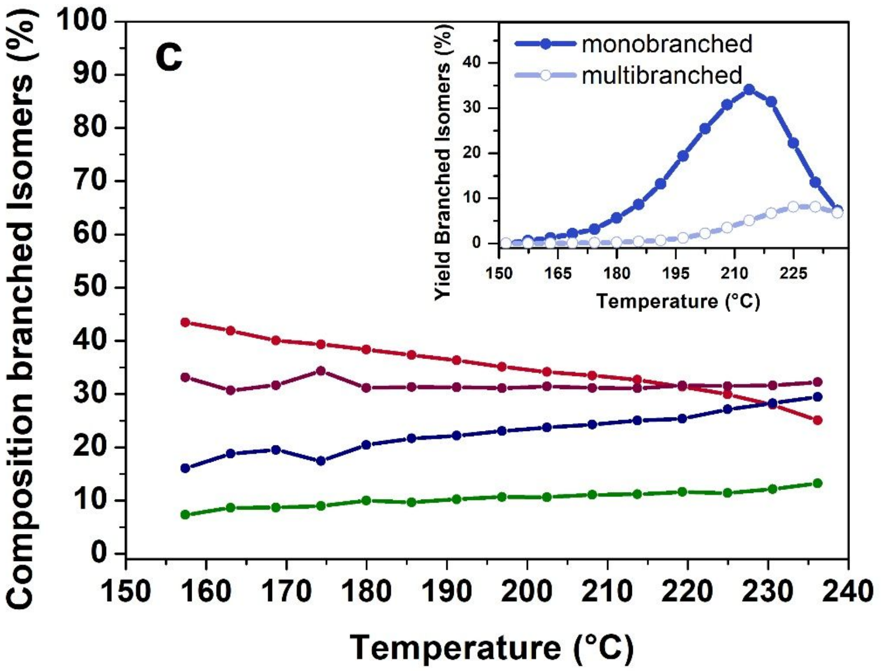

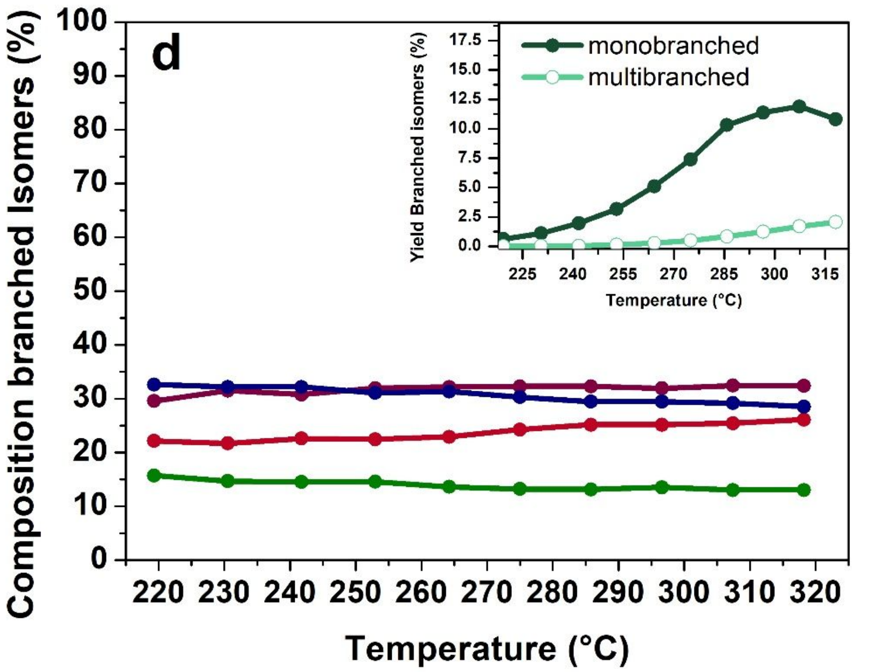

Figure S 8. *Composition of mono-branched isomerization products of n-decane converted by reference H-ZSM-5 (a) H-mM-Z1(b), H-mM-Z2 (c), H-Al-MCM-41 (d): 2-metylnonane (red lines); 3-metylnonane (purple lines); 4-metylnonane (blue lines); 5-metylnonane (green lines). Inset: comparison between yields of mono-branched (dark lines) and multi-branched (light lines) isomers.*

In 10-membered ring zeolites such as MFI, shape selectivity influences the composition of skeletal isomers from n-decane. Formation of ethyl branched C10 isomers (or substituents with higher carbon number) close to 3% is a clear indication of the presence of a 10MR framework, and in

particular, the methylbranching near 100% is typical of a H-ZSM-5 zeolite, as recorded for both H-mM-Z1 and H-mM-Z2. The steric limitation imposed by the very narrow 10MR channel suppresses the formation of cyclo-butyl alkylcarbenium intermediate or even larger ring transition states, which drives to the formation of ethyl branching, while the protonated cyclo-propyl (PCP) intermediates are favoured, bringing to methyl ramification. Another typical feature of 10MR frameworks is the suppression of multi-branched isomers, while mono-branched products are selectively favoured, again by steric limitation which imposes the PCP transition state. Moreover, the multi-branched isomers are generated only in a consecutive step to monobranching when the conversion is rising. This behaviour is shown in the insets of **Figure S8**, where the dark curves (mono-branched isomers yield) visibly overcome the light lines (multi-branched isomers yield) for all the reaction pathway in the presence of both H-mM-Z catalysts as it happens for the reference H-ZSM-5. **Figure S8** reports the distribution of mono-branched isomers until complete decane conversion: the main skeletal isomerisation product on H-ZSM-5, H-mM-Z1 and H-mM-Z2 is 2-methylnonane, along all the process, while the 5-methylnonane is always a minority product.[4]

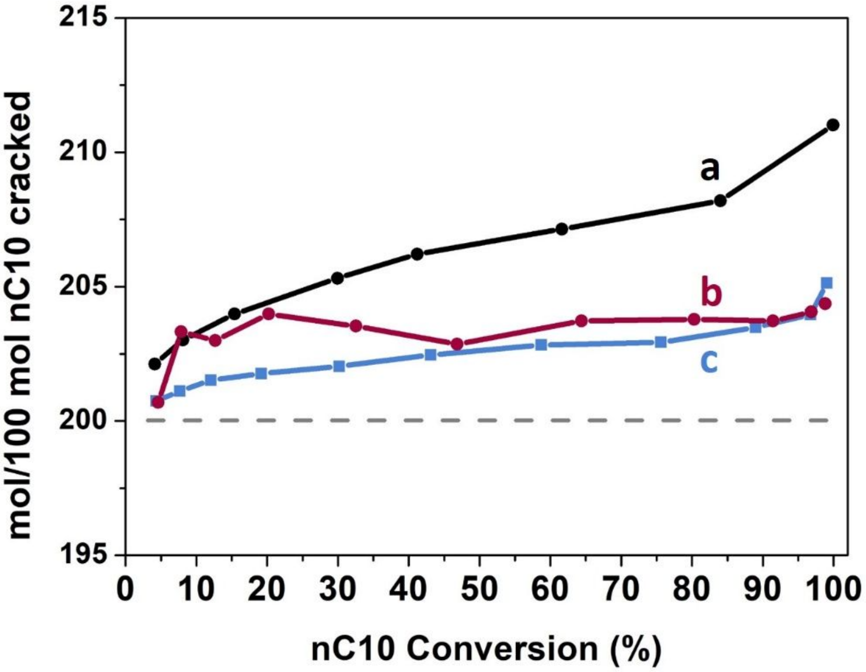

***Figure S 9**. Total amount of n-decane fractions per 100 moles of n-decane cracked at increasing conversion percentages over commercial H-ZSM-5 (a) H-mM-Z1 (b) and H-mM-Z2 (c).*

If only primary cracking occurs, the total sum of the molar yields of each fraction of cracked products out of 100 moles of feed n-decane should sum up to 200 mol per 100 mol n-decane cracked. A deviation from this marker number stands for secondary cracking reactions. Figure S9 shows the trends for the different catalysts. Both H-mM-Z (blue and red curves) show an almost constant sum of cracked product yields, very close to the 200 mol/100 mol cracking value, especially at the beginning of the reaction, until the isomerization reaction is overtaken by cracking. In contrast, on the H-ZSM-5 (black line) cracking generates substantially more than 200 mol fragments per 100 mol n-decane cracked.[5]

- ***References***

(1) Zecchina, A.; Bordiga, S.; Spoto, G.; Marchese, L.; Petrini, G.; Leofanti, G.; Radovan, M. Silicalite Characterization. 2. IR Spectroscopy of the Interaction of CO with Internal and External Hydroxyl Groups. *J. Phys. Chem.* **1992**, *96* (12), 4991–4997. https://doi.org/10.1021/j100191a048.

(2) Zecchina, A.; Spoto, G.; Bordiga, S. Vibrational Spectroscopy of Zeolites. In *Handbook of Vibrational Spectroscopy*; Chalmers, J. M., Ed.; John Wiley & Sons, Ltd: Chichester, UK, 2006; pp 301–332. https://doi.org/10.1002/0470027320.s7102.

(3) Bordiga, S.; Lamberti, C.; Bonino, F.; Travert, A.; Thibault-Starzyk, F. Probing Zeolites by Vibrational Spectroscopies. *Chem. Soc. Rev.* **2015**, *44* (20), 7262–7341. https://doi.org/10.1039/c5cs00396b.

(4) Martens, J. A.; Parton, R.; Uytterhoeven, L.; Jacobs, P. A.; Froment, G. F. Selective Conversion of Decane into Branched Isomers. *Appl. Catal.* **1991**, *76* (1), 95–116. https://doi.org/10.1016/0166-9834(91)80007-J.

(5) Verheyen, E.; Jo, C.; Kurttepeli, M.; Vanbutsele, G.; Gobechiya, E.; Korányi, T. I.; Bals, S.; Van Tendeloo, G.; Ryoo, R.; Kirschhock, C. E. A.; Martens, J. A. Molecular Shape-Selectivity of MFI Zeolite Nanosheets in n-Decane Isomerization and Hydrocracking. *J. Catal.* **2013**, *300* (2013), 70–80. https://doi.org/10.1016/j.jcat.2012.12.017.
